# Accumulation of autophagosomes confers cytotoxicity

**DOI:** 10.1074/jbc.M117.782276

**Published:** 2017-07-03

**Authors:** Robert W. Button, Sheridan L. Roberts, Thea L. Willis, C. Oliver Hanemann, Shouqing Luo

**Affiliations:** From the Peninsula Schools of Medicine and Dentistry, Institute of Translational and Stratified Medicine, University of Plymouth, Research Way, Plymouth PL6 8BU, United Kingdom

**Keywords:** autophagy, cell death, lysosome, mTOR complex (mTORC), neurodegenerative disease

## Abstract

Autophagy comprises the processes of autophagosome synthesis and lysosomal degradation. In certain stress conditions, increased autophagosome synthesis may be associated with decreased lysosomal activity, which may result in reduced processing of the excessive autophagosomes by the rate-limiting lysosomal activity. Thus, the excessive autophagosomes in such situations may be largely unfused to lysosomes, and their formation/accumulation under these conditions is assumed to be futile for autophagy. The role of cytotoxicity in accumulating autophagosomes (representing synthesis of autophagosomes subsequently unfused to lysosomes) has not been investigated previously. Here, we found that accumulation of autophagosomes compromised cell viability, and this effect was alleviated by depletion of autophagosome machinery proteins. We tested whether reduction in autophagosome synthesis could affect cell viability in cell models expressing mutant huntingtin and α-synuclein, given that both of these proteins cause increased autophagosome biogenesis and compromised lysosomal activity. Importantly, partial depletion of autophagosome machinery proteins Atg16L1 and Beclin 1 significantly ameliorated cell death in these conditions. Our data suggest that production/accumulation of autophagosomes subsequently unfused to lysosomes (or accumulation of autophagosomes) directly induces cellular toxicity, and this process may be implicated in the pathogenesis of neurodegenerative diseases. Therefore, lowering the accumulation of autophagosomes may represent a therapeutic strategy for tackling such diseases.

## Introduction

Macroautophagy (referred to as “autophagy” hereafter) is an intracellular degradation process that mediates the bulk clearance of long-lived and aberrant proteins, defective organelles, and certain pathogens. Autophagy provides a source of nutrition during periods of stress to promote healthy cell homeostasis and boost survival. Autophagy proceeds through the sequential nucleation and elongation of a double-membraned vesicle called an autophagosome, encapsulating a portion of the cytoplasm in the process. Autophagosomes then shuttle to lysosomes, whereupon the vesicles fuse, forming an autolysosome. The acidic pH and enzymatic action of hydrolases within the lysosome lead to the breakdown of the internal membranes of autophagosomes as well as the autophagosomes' contents (1). This clearance has been implicated in a diverse range of pathologies, including tumorigenesis, neurodegenerative disease, and infection, among others (2).

The various stages of autophagy each have their own set of regulators. The most well-characterized master controller of the process is the target of rapamycin complex (TOR,^2^ mTOR in mammals), which serves to repress autophagosome biogenesis and autophagy under basal and stimulation conditions. Upon the onset of certain stresses, when TOR is deactivated, autophagosome biogenesis and autophagy are initiated (3). Autophagosome formation is mediated by a dedicated machinery of autophagy-related (Atg) genes. Originally observed in yeast, the Atg family is highly conserved across species, and nearly 40 members have been identified. The sequential ULK1 and Beclin 1–Vps34 complexes are required to nucleate the developing autophagosome membrane (4, 5). Two ubiquitin-like conjugation reactions then elongate the membrane to the completed double-membraned autophagosome. In these reactions, different Atg proteins serve roles akin to the E1- and E2-like enzymes found in the ubiquitin-proteasome system. The first involves the conjugation of Atg12 to Atg5, facilitated by Atg7 (E1-like) and Atg10 (E2-like) (6). This Atg12–5 then binds non-covalently to Atg16 (7). In the second, LC3 is cleaved by Atg4 and conjugated to phosphatidylethanolamine via Atg7 (E1) and Atg3 (E2) (6, 8, 9), and the Atg5–12–Atg16 complex serves as an E3 ligase to stimulate LC3-phosphatidylethanolamine or LC3-II conjugation (10–12). LC3-II remains associated with autophagosome membranes following completion, making it a widely used marker for these vesicles (13). Interestingly, recent data suggest that although the ATG (autophagy-related gene) conjugation systems are critical for autophagosome completion, they may not be absolutely essential for the process (14, 15).

Once completed, autophagosomes and their cargo are shuttled to lysosomes for fusion and subsequent degradation. Multiple complexes appear to facilitate autophagosome–lysosome fusion. These include the lysosome-associated membrane proteins 1 and 2 (LAMP-1 and LAMP-2), the two proteins required for the trafficking of lysosomes and autophagosome–lysosome fusion (16, 17). The SNARE syntaxin-17 (STX-17) is recruited to completed autophagosomes and interacts with SNAP29 and the lysosomal SNARE VAMP-7, serving as a tether between autophagosomes and lysosomes (18). Components of the HOPS complex, such as VPS16, VPS33A, and VPS39, have also been shown to interact with STX-17 and aid this process (19, 20). As such, genetic ablation of any of these proteins results in a blockade to autophagy flux, causing accumulations of non-fused autophagosomes. Lysosomal degradation is dependent on an acidic pH (21), meaning that chemical agents like bafilomycin-A1 (Baf) and chloroquine (CQ), which reduce acidity, also cause a disruption to autophagy flux (22, 23).

Currently, autophagy is largely believed to function as a pro-survival process due to its critical role in cellular energy and nutrition homeostasis, and autophagy inhibition compromises cell viability (24). Various stress conditions, such as oxidation and toxic protein aggregation, often concurrently induce both compromised autolysosomal activity and increased autophagosome synthesis (25). In these conditions, the excessive autophagosomes resulting from the elevated synthesis cannot be processed by the rate-limiting compromised lysosomal activity. Thus, the excessive autophagosomes are non-fused, and their synthesis/accumulation in the conditions may be a futile process for autophagy. Although defective autolysosomal activity is well recognized to exacerbate cell survival (26), it has not been investigated whether accumulation of autophagosomes, which represents the synthesis of autophagosomes that subsequently fail to fuse with lysosomes, also contributes to the cell toxicity in stress conditions. In this study, we exploited genetic and chemical inhibition approaches to dissect the effects of increased autophagosome formation or lysosomal dysfunction on cell viability, both alone and in combination with one another (the latter strategy representing accumulation of autophagosomes). We found that defects in autophagosome–lysosome fusion or lysosomes alone do not induce sufficient cellular toxicity. However, increased autophagosome synthesis cooperates with defective lysosomal activity to synergistically induce cell toxicity/death. These data, for the first time, highlight that accumulation of autophagosomes directly exerts cellular toxicity during late-stage autophagy inhibition. Our data also suggest that autophagosome synthesis appears to be required for the toxicity caused by defects in autophagosome–lysosome fusion or lysosomes. Importantly, we confirm that partial depletion of autophagosome machinery proteins indeed alleviates cell death in cells expressing toxic mutant huntingtin and α-synuclein. Therefore, our findings may be implicated in the pathogenesis and therapies of neurodegenerative diseases.

## Results

### Simultaneous ablation of mTOR and STX-17 synergistically causes production/accumulation of non-fused autophagosomes

Previously, we reported (27) that PI3K/mTOR dual kinase inhibitors induce cell death as well as stimulate autophagosome biogenesis while also causing a defect in autophagosome–lysosome fusion. We reasoned that although autophagy is usually a pro-survival process, increased autophagosome biogenesis may be a contributing factor to toxicity in such contexts. Because in these circumstances there is disruption to autophagosome–lysosome fusion, the autophagosomes cannot be processed. This therefore means that they can be considered as non-fused autophagosomes, which yield no survival benefits and may instead pose cell toxicity. We began by testing this hypothesis via the use of the autophagy inducer rapamycin (Rap) and the lysosomal de-acidifier CQ. Whereas single use of Rap caused no significant alteration to cell viability, CQ did produce a notable decline. Importantly, this viability loss could be exacerbated further by combining the two drugs, suggesting that increased autophagosome biogenesis in periods of lysosomal failure (accumulation of autophagosomes) may exert cell toxicity (supplemental Fig. S1).

**Figure 1.**
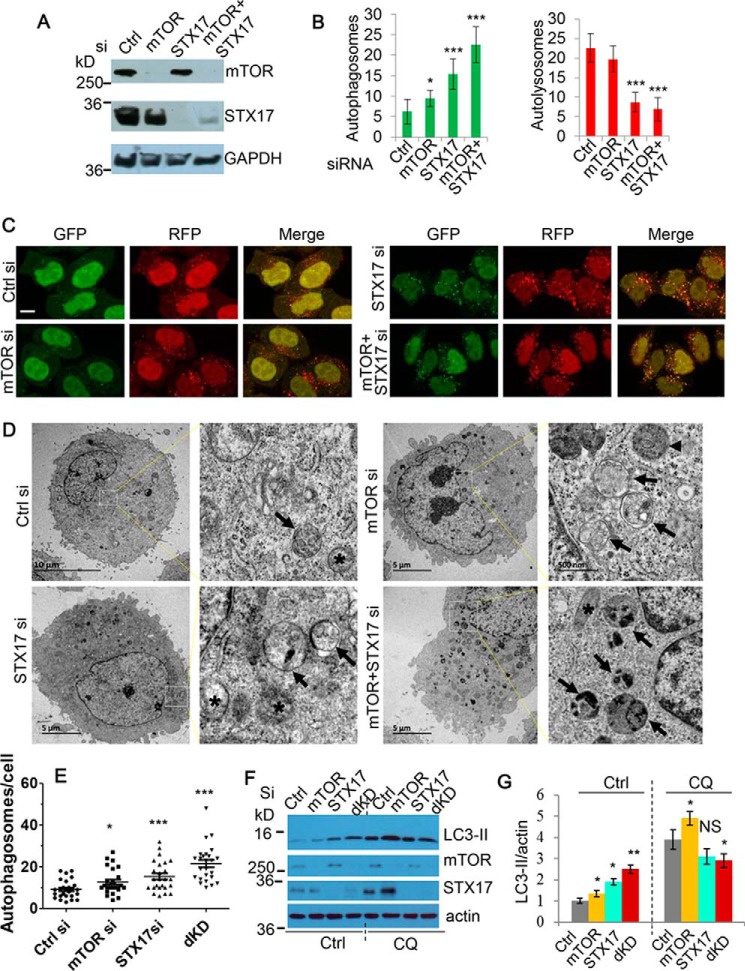
**Establishment of a model for production/accumulation of non-fused autophagosomes by simultaneous ablation of mTOR and STX-17.** *A*, immunoblot was used to confirm the knockdown efficiency of mTOR and STX-17 siRNA transfection in HeLa cells over 72 h. *B*, mRFP-GFP-LC3 stably expressing HeLa cells were transfected with control, mTOR, STX-17, or mTOR + STX-17 siRNA for 72 h. The number of autophagosomes (*green vesicles*) and autolysosomes (*red vesicles* minus *green vesicles*) was assessed (*n* = 20 cells/condition). Data are shown as mean ± S.D. (*error bars*). *, *p* < 0.05; ***, *p* < 0.001. *C*, representative confocal images for the cells in *B* were collected. *Scale bar*, 20 μm. *D*, HeLa cells were transfected with siRNAs as indicated for 48 h. Images were acquired by TEM microscopy. *Black arrows*, indicate autophagosomes; *arrowheads*, autolysosomes; *asterisks*, mitochondria. *E*, quantification of autophagosomes in 25–26 cells for each condition. *dKD*, dual knockdown (mTOR/STX-17 siRNA). *, *p* < 0.05; ***, *p* < 0.001. *F*, HeLa cells were transfected as in *B*, with vehicle or CQ (25 μm) treatment for the last 24 h. Cell lysates were subjected to immunoblotting and probed with the indicated antibodies. *G*, LC3-II levels in *F* were quantified *versus* loading control (actin). Data are shown as mean -fold change ± S.D. (*n* = 3). *, *p* < 0.05; ***, *p* < 0.001; *NS*, not significant.

To further test whether the accumulation of autophagosomes exerts cytotoxicity, we next aimed to establish a model that represents the conditions for the formation of non-fused autophagosomes by using siRNA as a more specific genetic approach. We thus targeted the negative regulator of autophagy-mTOR (3) and one of the key autophagosome–lysosome fusion mediators, STX-17 (18), using siRNA, to induce autophagosome synthesis and disrupt degradation, respectively. To ensure that this strategy exerts the desired effect on autophagosomes, we first utilized cells stably expressing mRFP-GFP-LC3. This system enables the distinction between non-fused autophagosomes and autolysosomes, because the acidic lysosomal pH quenches GFP (28). Consistent with our expectations, siRNA knockdown of STX-17 led to an increase in non-fused autophagosomes, which was exacerbated further when coupled with mTOR knockdown (Fig. 1, *A–C*). To fortify these LC3 counts, we also assessed numbers of autophagic vesicles by EM. These results concurred with the mRFP-GFP-LC3 data, with synergistic knockdown of mTOR and STX-17 yielding a greater number of autophagosomes than either siRNA alone (Fig. 1, *D* and *E*). In addition, immunoblot analysis of LC3-II levels showed similar effects. Notably, co-treatment with the lysosomal inhibitor CQ only efficiently elevated LC3-II in the control or mTOR single knockdowns, consistent with the role of STX-17 in mediating autophagosome–lysosome fusion (Fig. 1, *F* and *G*). Together, these results highlight the dual knockdown of mTOR and STX-17 as a valid method to generate accumulation of autophagosomes.

### Simultaneous ablation of mTOR and STX-17 synergistically causes cell viability loss

**Figure 2.**
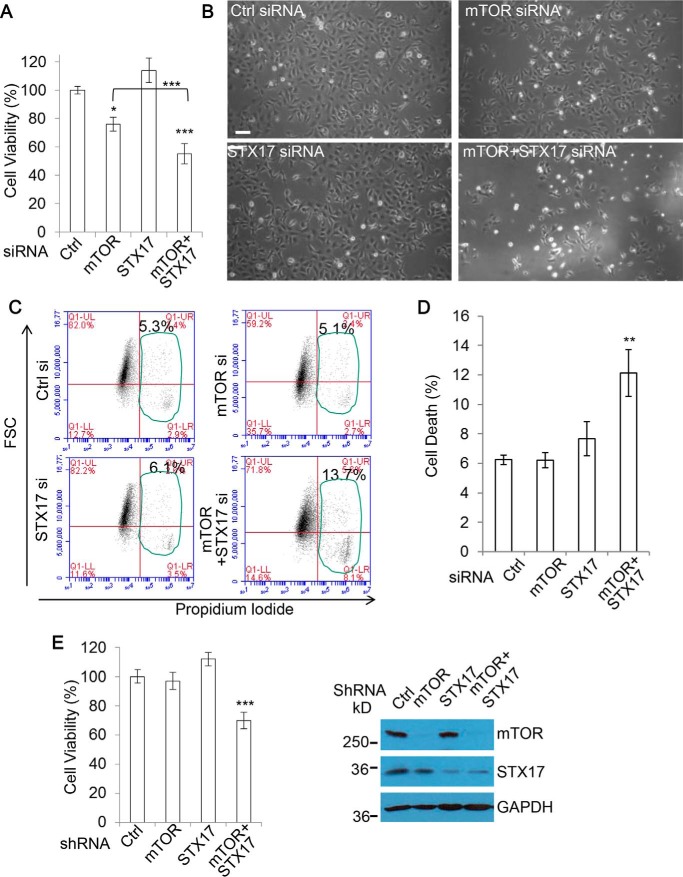
**Dual mTOR/STX-17 knockdown causes cell viability loss.** *A*, HEK293 cells were transfected with control, mTOR, STX-17, or mTOR + STX-17 siRNA for 72 h, and cell viability was measured with an MTT assay (*n* = 6 cells/condition). Data are shown as mean ± S.D. (*error bars*). *, *p* < 0.05; ***, *p* < 0.001. *B*, matching phase-contrast images were acquired. *Scale bar*, 100 μm. *C*, HEK293 cells were transfected as in *A*, and cell death was measured using propidium iodide staining in flow cytometry (*n* = 6/treatment). *D*, results from *C* are shown as mean ± S.D. **, *p* < 0.01. *E*, HEK293 cells were transfected with control, mTOR, STX-17, or mTOR + STX-17 shRNA for 72 h, and cell viability was measured (*n* = 6 cells/condition). Data are shown as mean ± S.D. ***, *p* < 0.001. Knockdown efficiency was confirmed by immunoblotting.

Interestingly, combination of mTOR and STX-17 siRNA led to a synergistic decline in cell viability, greater than the viability loss caused by treatment with mTOR siRNA or STX-17 siRNA alone (Fig. 2, *A* and *B*). With the combination treatments proving the most detrimental to the cells, these data suggest that increased autophagosome synthesis, in addition to lysosomal defect, is required for sufficient cytotoxicity. Notably, whereas depletion of STX-17 alone did not cause any significant changes, single mTOR knockdown did cause a decrease in viability. This is not surprising, because mTOR is a master regulator of growth and proliferation, two factors that are also encompassed by viability measures in addition to cell health and survival. To account for this, we measured proliferation under the same conditions and corrected the viability against the proliferation (supplemental Fig. S2). Importantly, whereas the viability decrease was mostly lost in mTOR single siRNA treatment after proliferation factor was excluded, a significant decline was still evident for the combination treatment by mTOR siRNA and STX-17 siRNA after the exclusion of proliferation factor, indicating a significant degree of cell toxicity and/or death in the dual knockdown conditions (supplemental Fig. S2). We also confirmed that levels of cell death detectable by propidium iodide staining in flow cytometry were increased in the conditions (Fig. 2, *C* and *D*). Of note, the cell death rate is lower than that of viability loss, given that it only accounts for a portion of the toxicity. To ensure that the effects on viability loss from STX-17 siRNA knockdown were via its inhibition of autophagosome–lysosome fusion, and not some as yet unreported off-target roles, we also used mTOR shRNA and STX-17 shRNA for the experiments. Fig. 2*E* shows that mTOR/STX-17 shRNA dual knockdown consistently induced cytotoxicity. These data suggest that autophagosome biogenesis stimulated by mTOR knockdown is important to sensitize cells to lysosomal defects or that formation/accumulation of non-fused autophagosomes can directly exert cytotoxicity.

We fortified these experiments with some additional drug strategies. We have previously shown the dual PI3K/mTOR inhibitor PI-103 to stimulate autophagosome formation while blocking degradation to a degree (27), which can be exacerbated further by coupling it with lysosomal the de-acidifier CQ or Baf. With these drug treatments, we again observed that whereas single administration of either agent caused a significant decline in viability, the effect could be exacerbated dramatically by using the two in combination (supplemental Fig. S3, *A* and *B*). Also, total loss of viability was achievable at much lower concentrations of PI-103 when used with Baf (supplemental Fig. S3*C*). As well as assessing changes to cell viability, we also measured the cytotoxicity under conditions of autophagosome synthesis with lysosomal blockade. Consistent with our previous measurements, we found that the toxicity associated with lysosomal inhibition could be exacerbated by coupling it with an autophagosome synthesis inducer (Rap) (supplemental Fig. S3*D*). Together, these results further support a toxic role for accumulation of autophagosomes achieved through a combination of elevated autophagosome synthesis coupled with decreased degradation.

### Increased accumulation of autophagosomes through diverse targets widely induces cytotoxicity

**Figure 3.**
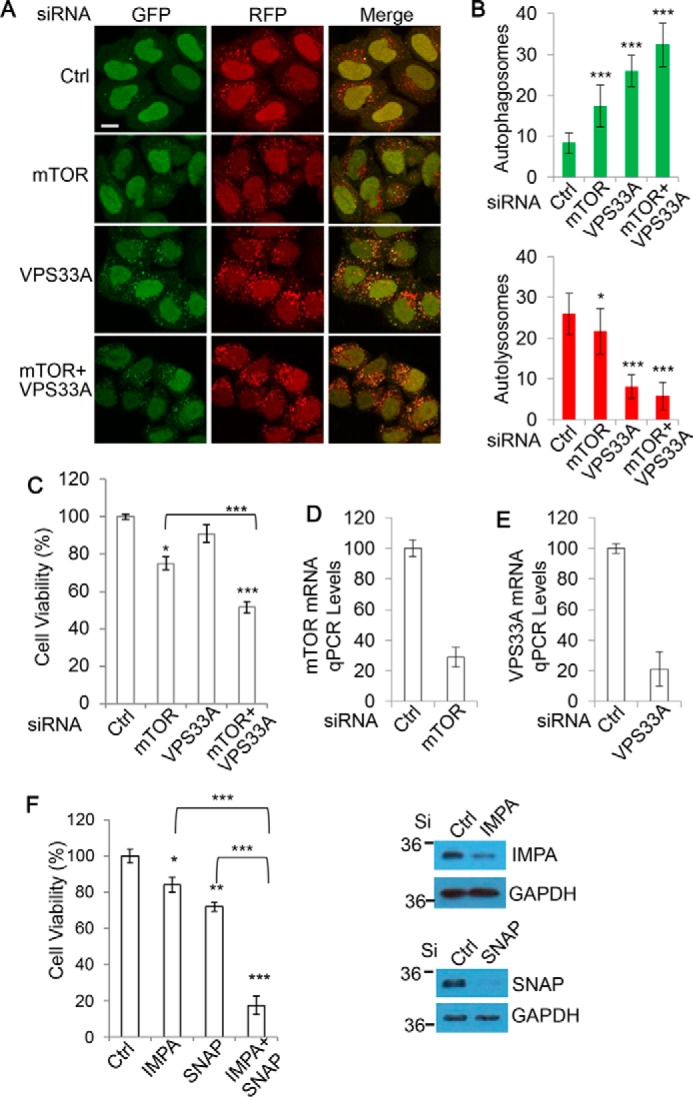
**Increased synthesis/accumulation of non-fused autophagosomes through diverse targets widely induces cytotoxicity.** *A*, HeLa cells stably expressing mRFP-GFP-LC3 were transfected with control, mTOR, Vps33A, or mTOR + Vps33A siRNA for 72 h. Cells were fixed, and confocal images were collected. *Scale bar*, 20 μm. *B*, the number of autophagosomes (*green vesicles*) and autolysosomes (*red vesicles* minus *green vesicles*) from *A* was assessed (*n* = 20 cells/condition). Data are shown as mean ± S.D. (*error bars*). *, *p* < 0.05; ***, *p* < 0.001. *C*, HEK293 cells were transfected as in *A*, and viability was measured with an MTT assay (*n* = 6 cells/condition). Data are shown as mean ± S.D. *, *p* < 0.05; ***, *p* < 0.001. *D*, knockdown efficiency for the mTOR siRNA was confirmed with qPCR. *E*, knockdown efficiency for the VPS33A siRNA was confirmed with qPCR. *F*, HEK293 cells were transfected with control, IMPA, SNAP29 (*SNAP*), or IMPA + SNAP siRNA for 72 h. Cell viability was measured with an MTT assay (*n* = 5 cells/condition). Data are shown as mean ± S.D. *, *p* < 0.05; **, *p* < 0.01; ***, *p* < 0.001. Knockdown efficiency was confirmed with immunoblotting.

To ensure that the effects on viability loss from mTOR/STX-17 dual knockdown were attributable to the increased formation of autophagosomes coupled with inhibition of autophagosome–lysosome fusion, we aimed to replicate the toxicity with alternative targets. Vps33A is a member of the HOPS complex and also has been shown to be necessary for autophagosome–lysosome fusion (19). We confirmed this with cells stably expressing mRFP-GFP-LC3. As with STX-17 knockdown, ablation of Vps33A caused a blockade to autolysosome formation, with an increase in the number of non-fused autophagosomes (Fig. 3, *A* and *B*). Importantly, this could be exacerbated by pairing Vps33A siRNA with mTOR siRNA (Fig. 3, *A* and *B*). This combination effect was also translated to cell viability. Simultaneous Vps33A and mTOR knockdown consistently led to a significant decline in cell viability, to a degree comparable with that with mTOR and STX-17 siRNA combinations (Fig. 3*C*). mTOR and Vps33A knockdown was confirmed by qPCR (Fig. 3, *D* and *E*). Other important mediators of autophagosome–lysosome fusion are the LAMP proteins (29, 30). Therefore, LAMP1/2 double knock-out cells represent an alternative model for autophagosome clearance failure. Notably, we found the viability of LAMP double knock-out cells to be more sensitive to exposure to starvation conditions, a *bona fide* stimulator of autophagosome synthesis, than the wild type (supplemental Fig. S4*A*). These data further indicate that production of autophagosomes is toxic under periods of lysosomal failure.

Given that mTOR also regulates other cellular pathways in addition to autophagosome synthesis, we wanted to ensure that our toxicity measurements were not attributable to additional roles of mTOR. Therefore, our attention turned to utilizing mTOR-independent methods to stimulate autophagosome synthesis. Several mTOR-independent mechanisms of autophagy activation have been identified, including via the inositol signaling pathway. Studies have shown that reductions in free inositol lead to enhanced autophagosome synthesis (31). For this reason, we opted to target inositol monophosphatase 1 (IMPA) with siRNA as a means to induce autophagosome generation without disrupting mTOR. Consistent with our expectations, we confirmed IMPA knockdown to yield an increase in autophagosome numbers, which could be elevated further when coupled with CQ (supplemental Fig. S4, *B* and *C*). With IMPA appearing to be a suitable target for autophagosome synthesis, we explored whether combining it with lysosomal inhibition could cause toxicity as before. We paired IMPA with SNAP29, which has been demonstrated to be important for autophagosome–lysosome fusion (18), as shown in supplemental Fig. S4, *B* and *C*. Importantly, we found that whereas knockdown of either IMPA or SNAP29 alone led to a modest reduction in cell viability, this could be dramatically enhanced by combining the treatments (Fig. 3*F*).

Chemical strategies to induce autophagosome synthesis independent of mTOR have also been developed. We selected rilmenidine (Ril), which stimulates autophagosome synthesis by reducing cAMP levels (32), to fortify our IMPA observations. Importantly, whereas Ril itself caused no viability decline, it did synergize with CQ to cause significant cell viability loss (supplemental Fig. S4*D*). Together, these data suggest that mTOR-independent stimulation of autophagosome synthesis synergistically induces cytotoxicity when coupled with lysosomal defect, much like mTOR inhibition. These complementary data strongly suggest that increased autophagosome synthesis induces cytotoxicity in the case of autophagosomes not being processed by lysosomes.

### Autophagosome synthesis is required for the toxicity associated with accumulation of autophagosomes

**Figure 4.**
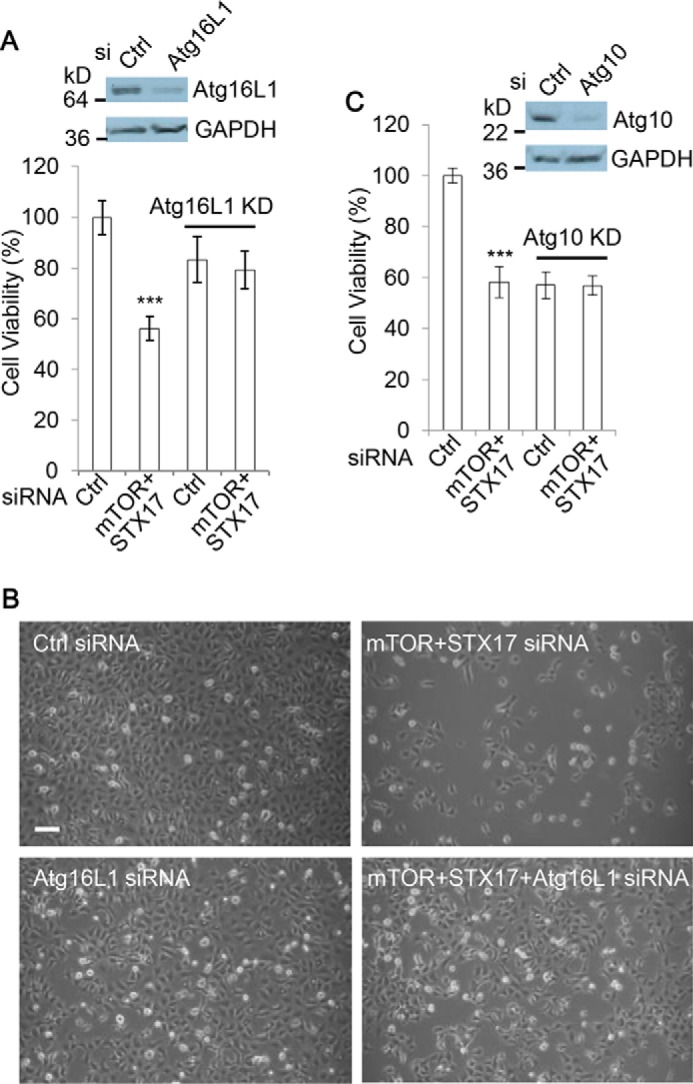
**Autophagosome synthesis is necessary for the viability loss by dual mTOR/STX-17 knockdown.** *A*, HEK293 cells were transfected with control or mTOR + STX-17 siRNA in the absence or presence of Atg16L1 siRNA, as indicated, for 72 h. Cell viability was measured with an MTT assay (*n* = 6 cells/condition). Data are shown as mean ± S.D. (*error bars*). ***, *p* < 0.001. Knockdown efficiency was confirmed by immunoblotting. *B*, matching phase-contrast images were acquired. *Scale bar*, 100 μm. *C*, HEK293 cells were transfected with control or mTOR + STX-17 siRNA in the absence or presence of Atg10 siRNA, as indicated, for 72 h. Cell viability was measured with MTT assay (*n* = 6 cells/condition). Data are shown as mean ± S.D. ***, *p* < 0.001. Knockdown efficiency was confirmed by immunoblotting.

Our data suggest that dual mTOR/STX-17 knockdown toxicity may be attributable to non-fused autophagosome formation. Therefore, we next asked whether a reduction in autophagosome biogenesis can alleviate mTOR/STX-17 dual knockdown toxicity. As such, we inhibited autophagosome synthesis through the use of siRNAs targeting Atg16L1 (Fig. 4, *A* and *B*) or Atg10 (Fig. 4*C*), two key mediators of autophagosome elongation. Intriguingly, we found that following the depletion of autophagosome formation, the viability loss induced by mTOR/STX-17 knockdown no longer occurred (Fig. 4, *A–C*). Similar to this, depletion of Atg16L1 also caused a reduction in toxicity associated with IMPA/SNAP29 knockdown (supplemental Fig. S5*A*). These data suggest that continued autophagosome synthesis is required for the loss of cell health under these conditions.

To complement these experiments, we also utilized autophagy chemical inhibition strategies to see whether these could alleviate the relevant viability losses. 3-Methyladenine (3MA) is a pan-PI3K inhibitor and thus can inhibit autophagosome synthesis due to the role of the class III PI3K in the process (33, 34). Notably, we found the addition of 3MA to greatly reduce the viability loss associated with PI-103 treatment (supplemental Fig. S5*B*). We also utilized an autophagosome synthesis-null cell line, Atg16L1 KO MEFs. In these cells, the depletion of autophagosome synthesis led to a significant reduction in the toxicity associated with both PI-103 and Baf, with the most notable rescue arising from the combination treatments with the two drugs (supplemental Fig. S5*C*).

From these findings, we conclude that when lysosomal function is blocked, autophagosome synthesis or accumulation of autophagosomes, a futile action of autophagosome synthesis, exerts cellular toxicity. The toxicity could be relevant in various pathological conditions. For instance, dementia diseases with toxic protein aggregates can be associated with compromised lysosomal activity (35), and the toxicity stress induced by this could in turn also increase autophagosome synthesis, leading to accumulation of autophagosomes.

### Accumulation of autophagosomes causes cell viability loss independent of apoptosis and necroptosis

**Figure 5.**
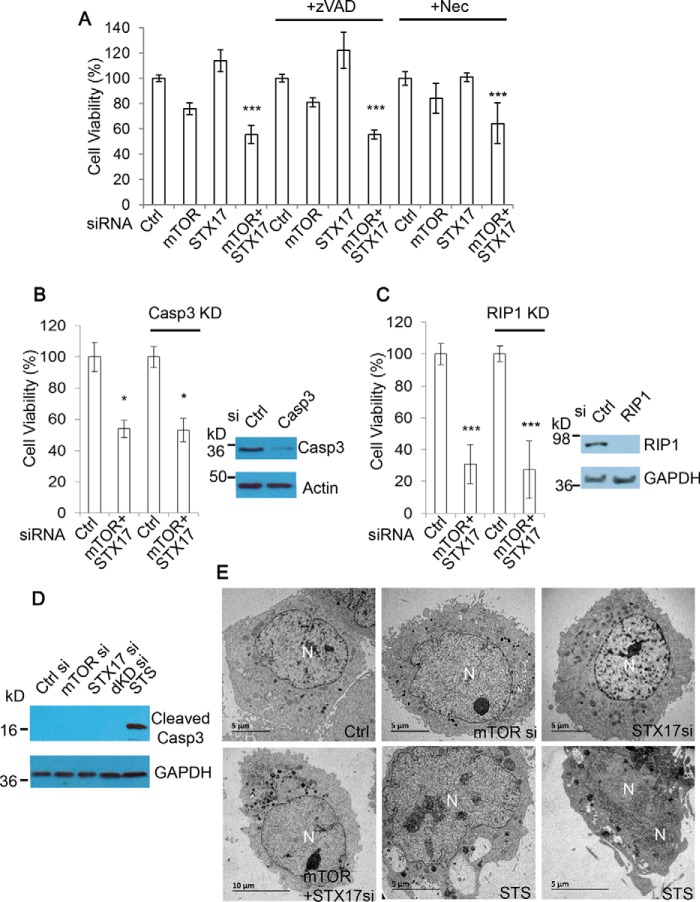
**Accumulation of autophagosomes causes cell viability loss independent of apoptosis and necroptosis.** *A*, HEK293 cells were transfected with control, mTOR, STX-17, or mTOR + STX-17 siRNA for 48 h and then treated with pan-caspase inhibitor Z-VAD-fmk (*zVAD*) (20 μm) or necroptosis inhibitor Nec (20 μm), as indicated, for a further 24 h. Cell viability was then measured with MTT assay (*n* = 6 cells/condition). Data are shown as mean ± S.D. (*error bars*). ***, *p* < 0.001. *B*, HEK293 cells were transfected with control or mTOR + STX-17 siRNA in the absence or presence of caspase-3 siRNA, as indicated, for 72 h. Cell viability was measured with an MTT assay (*n* = 6 cells/condition). Data are shown as mean ± S.D. *, *p* < 0.05. Knockdown efficiency was confirmed by immunoblotting. *C*, HEK293 cells were transfected with control or mTOR + STX-17 siRNA in the absence or presence of RIP1 siRNA, as indicated, for 96 h. Cell viability was measured with MTT assay (*n* = 6 cells/condition). Data are shown as mean ± S.D. ***, *p* < 0.001. Knockdown efficiency was confirmed by immunoblotting. *D*, HeLa cells were transfected with control, mTOR, STX-17, or mTOR + STX-17 (*dKD*) siRNA for 72 h and with the apoptosis inducer STS (1 μm) for 24 h as a positive control. Cell lysates were analyzed by immunoblot and probed with the indicated antibodies. *E*, HeLa cells were treated with siRNAs as indicated for 48 h or STS (1 μm) for 24 h. Images were acquired with TEM.

We next aimed to uncover the potential mechanism through which autophagosome accumulation toxicity occurs. Apoptosis and necroptosis are the two main routes of cell death, so we examined whether they are involved in autophagosome accumulation toxicity. Apoptosis is inhibited by the pan-caspase inhibitor benzyloxycarbonyl-VAD-fluoromethyl ketone (Z-VAD-fmk) (36), and necroptosis can be blocked by necrostatin-1 (Nec) (37), a selective inhibitor of RIP1 that is essential for RIP1-RIP3-dependent necroptosis (38–40). We tested whether the addition of these inhibitors could rescue cell viability loss caused by mTOR/STX-17 dual knockdown. We observed that mTOR/STX-17–induced cell viability loss was largely unaffected by either Z-VAD-fmk or Nec treatment (Fig. 5*A*), suggesting that this toxicity is independent of apoptosis and necroptosis. We fortified this observation by employing genetic strategies to ablate apoptosis or necroptosis, with siRNAs against caspase-3 (an apoptosis executioner (41)) to block apoptosis or against RIP1 for necroptosis inhibition. Consistently, disruption to either of the pathways did little to alleviate the viability loss with mTOR/STX-17 knockdown (Fig. 5, *B* and *C*). These results further confirm that apoptosis and necroptosis are not major determinants for the toxicity caused by accumulation of autophagosomes when autophagosome–lysosome fusion is compromised.

We also examined whether the addition of these inhibitors could rescue cell death associated with PI-103 and CQ treatment. Indeed, the massive extent of cell death caused by the combination of PI-103 and CQ was not reduced by either Z-VAD-fmk or Nec, further suggesting this lethality to be largely independent of apoptosis and necroptosis (supplemental Fig. S6, *A* and *B*). Additionally, we did not detect any caspase-3 cleavage fragments (which are a hallmark of apoptosis (41)) in the cells with mTOR/STX-17 treatment (Fig. 5*D*). TEM images show that the cells treated with either the single siRNA knockdown or mTOR/STX-17 siRNA dual knockdown do not show a damaged or fragmented nuclear morphology, as seen in the cells treated with the apoptosis inducer staurosporine (Fig. 5*E*).

### Accumulation of autophagosomes causes an energy deficit and elevates reactive oxygen species (ROS)

**Figure 6.**
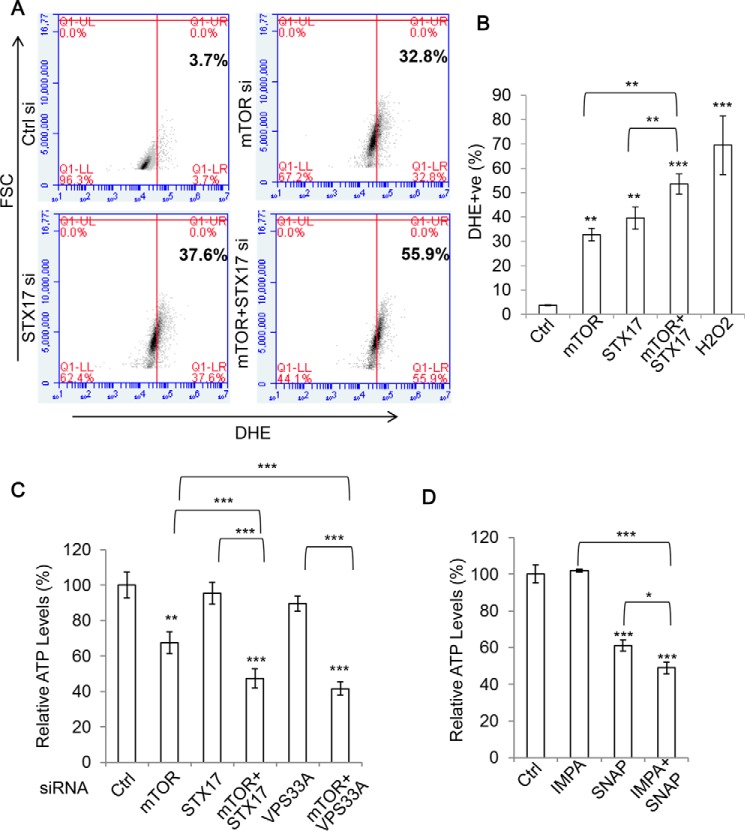
**Accumulation of autophagosomes causes an increase in ROS while depleting ATP levels.** *A*, HEK293 cells were transfected with control, mTOR, STX-17, or mTOR + STX-17 siRNA for 72 h, and ROS levels were measured in flow cytometry via dihydroethidium (*DHE*) staining (*n* = 3 cells/condition). *B*, data from *A* are shown as mean ± S.D. (*error bars*). Treatment with 1 mm H_2_O_2_ for 30 min was used as a positive control. **, *p* < 0.01; ***, *p* < 0.001. *C*, HEK293 cells were treated with control, mTOR, STX-17, mTOR + STX-17, VPS33A, or mTOR + VPS33A siRNA as indicated for 72 h, and ATP levels were measured via an ATP assay kit (Promega) (*n* = 6 cells/condition). Data are shown as mean ± S.D. **, *p* < 0.01; ***, *p* < 0.001. *D*, HEK293 cells were treated with control, IMPA, SNAP29 (*SNAP*), or IMPA + SNAP siRNA for 72 h, and ATP levels were measured as in *C* (*n* = 6/condition). Data are shown as mean ± S.D. *, *p* < 0.05; ***, *p* < 0.001.

Because apoptosis and necroptosis did not seem to influence the loss in viability caused by the accumulation of autophagosomes, we turned to further elucidating the mechanisms underlying this effect. Because autophagy is important in the removal of various intracellular toxins, we reasoned that accumulation of autophagosomes could result in the harmful persistence of certain agents. One such example is ROS, with autophagy providing protection by their direct removal as well as eliminating their source, defective mitochondria. Indeed, we observed a significant increase in overall ROS levels in mTOR and STX-17 siRNA–transfected cells, both over control and single siRNA treatment, suggesting that these may contribute to the toxicity (Fig. 6, *A* and *B*). Interestingly, a subpopulation of cells, particularly STX-17/mTOR knockdown cells, that undergoes pronounced ROS production exhibits significant cell size increases indicated by forward-scattered light (FSC) counts (Fig. 6*A*). This suggests that the enlargement of cell size is associated with ROS production. Currently, little is known about how cell size homeostasis is regulated. However, evidence suggests that ROS production concurs with cell size increase during cell senescence (42), and H_2_O_2_ treatment leads to cell enlargement, at least over an extended time frame (43). It would be interesting to reveal whether ROS is an upstream stimulus to positively regulate cell size in this case.

Another important lifeline that autophagy provides cells is an energy supply via the degradation and recycling of unnecessary materials. We explored whether this source was disrupted under our toxic knockdown treatments by assessing intracellular ATP levels. Despite a significant decrease in ATP levels with mTOR knockdown treatment (presumably due to the roles of mTOR in cell metabolism (44)) and a modest ATP reduction with STX-17 or VPS33A knockdown, the combination of mTOR and STX-17/VPS33A siRNA caused a marked and synergistic decline in the ATP levels (Fig. 6*C*). We found a similar effect to occur by coupling IMPA1 with SNAP29, highlighting this event as occurring independently of mTOR inhibition (Fig. 6*D*). Therefore, it seems that the energy deficit resulting from defective autophagy degradation also contributes to autophagosome accumulation toxicity. Under these conditions, the newly formed autophagosomes cannot be fused and prove futile. Autophagosome accumulation induction would only exert a further demand on the cell's energy stores through the continued production of non-fused autophagosomes, which ultimately proves toxic. Thus, the costs of autophagosome accumulation may be culpable for, or at least contribute to, the toxicity that we observe.

### Lowering accumulation of autophagosomes by partial depletion of autophagosome synthesis provides a rescue from aggregation-prone protein toxicity

**Figure 7.**
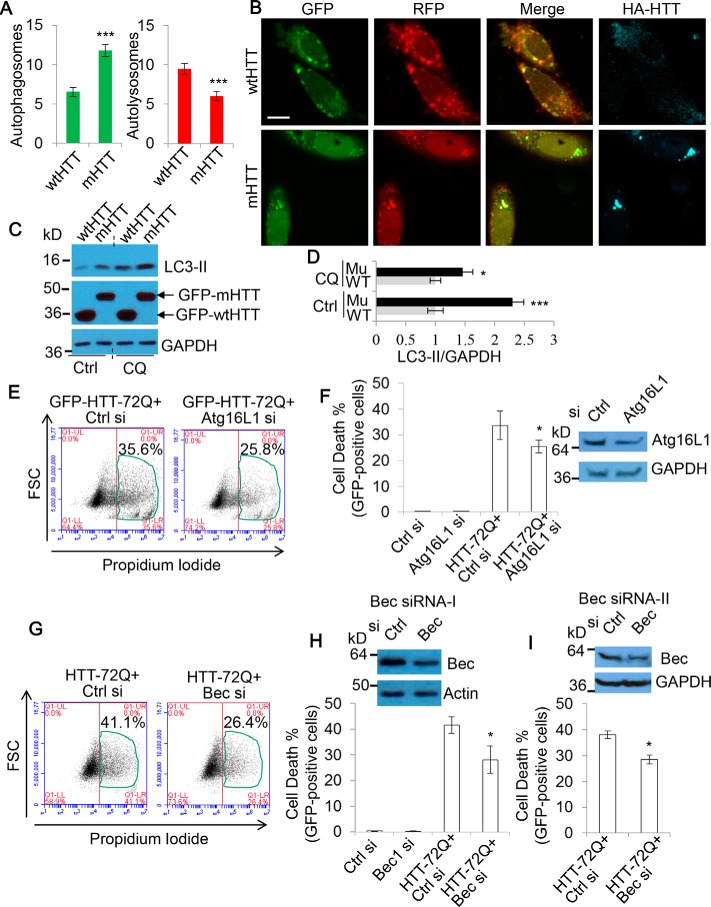
**Lowering accumulation of autophagosomes by partial depletion of autophagosome synthesis alleviates the toxicity of mutant huntingtin aggregation.** *A*, SK-N-SH cells were transfected with mRFP-GFP-LC3 and either HA-tagged WT wtHTT or mHTT for 48 h. Cells were then fixed, stained for HA, and imaged with confocal microscopy. *Scale bar*, 20 μm. The numbers of autophagosomes (*green vesicles*) and autolysosomes (*red vesicles* minus *green vesicles*) were calculated (*n* = 30 cells/experiment). Counts are shown as mean ± S.E. (*error bars*). ***, *p* < 0.001. *B*, shown are representative confocal images in *A*. Note that autophagosomes co-localize with mHTT aggregates. *C*, HeLa cells were transfected with GFP-tagged wtHTT or mHTT. After 72 h, cells were harvested (one set of cells were treated with 25 μm CQ for 8 h, as indicated, before harvesting). The cell lysates were subjected to SDS-PAGE and probed with the indicated antibodies. *D*, LC3-II levels were quantified over GAPDH. Data are shown as -fold change ± S.D. (*error bars*). (*n* = 3). *WT*, GFP-wtHTT; *Mu*, GFP-mHTT. *, *p* < 0.05; ***, *p* < 0.001. *E*, SK-N-SH cells were transfected with GFP-HTT exon 1 with 72Q (*HTT-72Q*) and either control or Atg16L1 siRNA (10 nm) for 48 h (to partially knock down Atg16L1). GFP-positive cells were gated, and cell death was measured by flow cytometry via propidium iodide staining (*n* = 6 cells/condition). Shown is the percentage of propidium iodide and GFP–double-positive cells/GFP-positive cells. *F*, data from *E* are shown as mean ± S.D. (*error bars*). *, *p* < 0.05. Partial knockdown was confirmed by immunoblotting. *G*, SK-N-SH cells were transfected with GFP-HTT-72Q and either control or Beclin 1 siRNA (20 nm) (*Bec siRNA-I*) for 48 h (to partially knock down Beclin 1). GFP-positive cells were gated, and cell death was measured as in *E* (*n* = 6 cells/experiment). Shown is the percentage of propidium iodide and GFP–double-positive cells/GFP-positive cells. *H*, data are shown as mean ± S.D. (*error bars*). *, *p* < 0.05. Partial knockdown was confirmed with immunoblotting. *I*, SK-N-SH cells were transfected as in *G* using an alternative Beclin 1 siRNA (Bec siRNA-II). Cell death was measured as before (*n* = 3 cells/experiment). Data are shown as mean ± S.D. (*error bars*). *, *p* < 0.05. Partial knockdown was confirmed with immunoblotting.

Many neurodegenerative diseases are associated with toxic protein aggregates, which have been shown to disrupt autophagy dynamics. Reports indicate that whereas the presence of these aggregates induces autophagosome synthesis, they also disrupt the degradation process (45–47). Therefore, our model of autophagosome accumulation–based toxicity seems relevant in these pathologies. We found the SK-N-SH neuroblastoma cell line to be sensitive to autophagosome accumulation inducing drug treatment (supplemental Fig. S1), so we opted to use these cells for our further autophagosome accumulation toxicity experiments. We aimed to assess whether autophagosome accumulation contributes to the toxicity of mutant huntingtin with expanded polyQ that causes Huntington's disease, in Huntington's disease cell models. To confirm that these cells are suitable for this study, we first assessed whether autophagosome–lysosome fusion was disrupted in neuronal SK-N-SH cells expressing mRFP-GFP-LC3, by comparing mutant huntingtin with 72Q (mHTT) *versus* wild-type huntingtin exon 1 with 21Q (wtHTT). We found mHTT to cause an increase in the number of non-fused autophagosomes compared with the wtHTT (Fig. 7, *A* and *B*), while not altering lysosomal pH or morphology (supplemental Fig. S7*A*). Because autophagosome numbers correlate with the levels of the autophagosome-associated protein LC3-II (1), we also examined LC3-II levels by immunoblotting. Fig. 7 (*C* and *D*) shows that LC3-II levels were markedly increased in mHTT-expressing cells when a lysosomal inhibitor was absent (Fig. 7*C*, *lanes 1* and *2*). After the treatment of the lysosomal inhibitor CQ for both wtHTT- and mHTT-expressing cells, although the -fold change in LC3-II levels was reduced, a significant increase was still observed in mHTT-expressing cells (Fig. 7*C*, *lanes 3* and *4*). The data (Fig. 7, *C* (*lanes* 3 and 4) and *D*) suggest that mHTT increases autophagosome formation, and the reduced LC3-II level change between wtHTT- and mHTT-expressing cells in the presence of CQ (Fig. 7*D*) suggests that the lysosomal pathway is defective in the cells expressing mHTT. We also confirmed that mHTT expression caused a significant increase in cell death (supplemental Fig. S7*B*). These data suggest that accumulation of autophagosomes occurs in cells expressing mHTT. Therefore, we asked whether depletion of components of the autophagosome formation machinery could alleviate the cell death resulting from mHTT.

Because autophagy is usually a pro-survival process, in many instances, strong ablation of the autophagy machinery can be detrimental to cell survival. Indeed, we observed this in our Atg10 and Atg16L1 knockdowns, which caused a degree of cell viability loss (Fig. 4, *A–C*). Therefore, to boost the possible translational relevance of our approach, we tested whether a partial knockdown to autophagosome synthesis using a lower siRNA dose (to ensure that cells retain sufficient autophagy activity) could yield protective benefits for the cells undergoing mHTT proteotoxicity. Following on from our earlier work (Fig. 4*A*), we started by targeting Atg16L1. Interestingly, we found that partial knockdown of Atg16L1 afforded a significant degree of protection from the cell death elicited from mHTT (Fig. 7, *E* and *F*).

To fortify this observation, we selected an alternative mediator of autophagosome formation, Beclin 1, the mammalian Atg6 homologue (48). Beclin 1 functions in two complexes, Complex I and Complex II, which influence autophagy at different stages. Complex I contains Vps34, Vps15, Beclin 1, and Atg14 and is important for autophagosome formation (5). However, Complex II, containing Beclin 1, UVRAG, and Rubicon, regulates the later process of autophagosome maturation (49, 50). Therefore, there is the risk that Beclin 1 depletion may also perturb autophagy flux, which according to our model would exacerbate the toxicity. To assess this, we investigated autophagy dynamics during Beclin 1 knockdown in cells expressing mRFP-GFP-LC3. Importantly, the numbers of autophagosomes were largely decreased following Beclin 1 depletion, whereas autolysosomes were less affected (supplemental Fig. S7, *C* and *D*). These data suggest that autophagosome formation is more severely affected by Beclin 1 depletion. As such, we explored whether Beclin 1 partial depletion could also provide a rescue from autophagosome accumulation toxicity in the cells with mHTT toxicity. Again, partial ablation of Beclin 1 caused a decline in cell death, to an extent even greater than that seen with Atg16L1 (Fig. 6 (*G* and *H*) and supplemental Fig. S7*E*). Importantly, we found this effect to be true for two different Beclin 1 siRNAs (Fig. 7*I*). Together, these results suggest that partial reduction in autophagosome formation alleviates autophagosome accumulation toxicity in mHTT-expressing cells, thereby providing a rescue from mHTT proteotoxicity.

**Figure 8.**
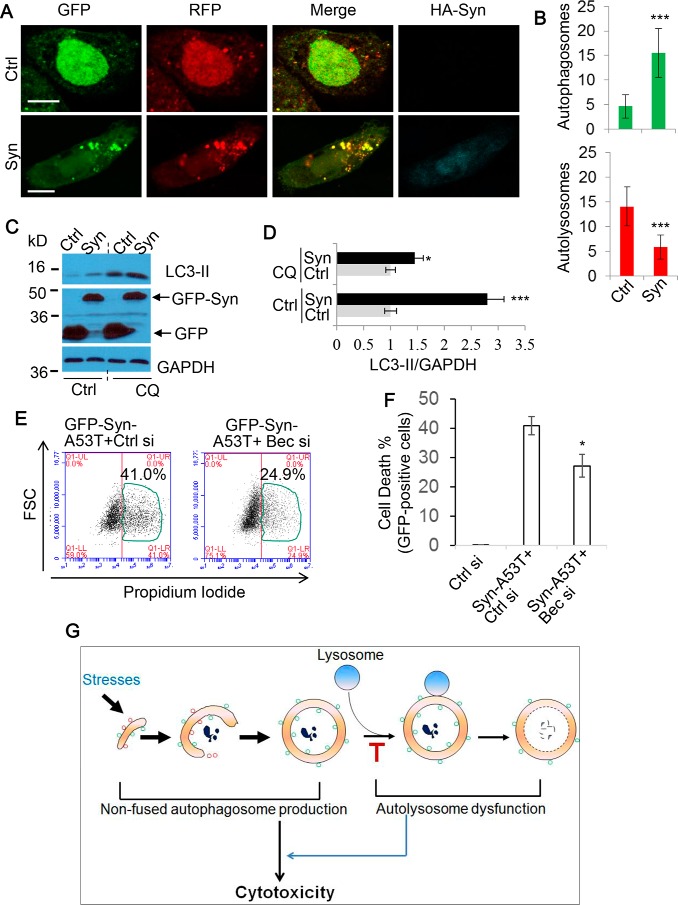
**Lowering accumulation of autophagosomes by partial depletion of autophagosome synthesis alleviates α-synuclein toxicity.** *A*, SK-N-SH cells were transfected with mRFP-GFP-LC3 and either control (*Ctrl*) or synuclein (*Syn*) for 48 h. Cells were fixed, stained for HA, and imaged with confocal microscopy. *Scale bars*, 20 μm. *B*, the numbers of autophagosomes (*green vesicles*) and autolysosomes (*red vesicles* minus *green vesicles*) were calculated (*n* = 20 cells/experiment). Counts are shown as mean ± S.D. (*error bars*). ***, *p* < 0.001. *C*, HeLa cells were transfected with GFP or GFP–α-synuclein. After 72 h, cells were harvested (one set of cells were treated with 25 μm CQ for 8 h before harvesting). The cell lysates were subjected to SDS-PAGE and probed with the indicated antibodies. *D*, the ratios of LC3-II/GAPDH were quantified. Data are shown as -fold change ± S.D. (*n* = 3). *, *p* < 0.05; ***, *p* < 0.001. *E*, SK-N-SH cells were transfected with GFP-Syn-A53T and either control or Beclin 1 siRNA (20 nm) for 48 h. GFP-positive cells were gated, and cell death was measured by flow cytometry via propidium iodide staining (*n* = 6 cells/condition). Shown is the percentage of propidium iodide and GFP–double-positive cells/GFP-positive cells. *F*, data from *E* are shown as mean ± S.D. *, *p* < 0.05. *G*, proposed model of autophagosome accumulation-based toxicity. During periods of stress, autophagosome synthesis will be promoted. However, if autophagosome–lysosome fusion is rendered dysfunctional, further non-fused autophagosome synthesis is futile to the cell because autophagy cannot be completed. Therefore, the synthesis of non-fused autophagosomes is detrimental to cell survival by causing more strain on energy levels as well as a failure to clear potentially harmful toxins.

Following our promising results with mHTT, we next tested whether this was the case for another toxic neurodegenerative protein, mutant α-synuclein. Accumulations of this protein are associated with Parkinson's disease, with several reports demonstrating it to disrupt autophagy flux (45–47). We confirmed this to be true in our experimental conditions by assessing autophagosome *versus* autolysosome numbers with mRFP-GFP-LC3–expressing cells (Fig. 8, *A* and *B*), again seeing an increase in non-fused or degraded vesicles, while not disrupting lysosomal pH (supplemental Fig. S8). Similarly, we also examined LC3-II levels by immunoblot under these conditions. Fig. 8 (*C* and *D*) shows that LC3-II levels were largely increased in α-synuclein–overexpressing cells without CQ treatment (Fig. 8*C*, *lanes 1* and *2*). With CQ treatment, a significant increase was still observed in α-synuclein–overexpressing cells (Fig. 8*C*, *lanes 3* and *4*). The data (Fig. 8*C*, *lanes 3* and *4*) suggest that α-synuclein overexpression increases autophagosome formation, and the reduced LC3-II increase in α-synuclein-overexpressing cells in the presence of CQ (Fig. 8*D*) confirms that the lysosomal pathway is defective in the cells overexpressing α-synuclein. These findings indicate that autophagosome accumulation also occurs in cells with α-synuclein aggregates.

With Beclin 1 partial depletion proving to be markedly protective against mHTT, we applied the same strategy to the cells expressing mutant α-synuclein. Consistently, we found that a reduction in Beclin 1 provided a significant degree of rescue from mutant α-synuclein toxicity (Fig. 8, *E* and *F*). Therefore, our model of autophagosome accumulation–based toxicity (Fig. 8*G*) may be relevant to neurodegenerative diseases in general.

## Discussion

In this study, we demonstrate that accumulation of autophagosomes induces cellular toxicity. Our data show that stimulation of autophagosome synthesis can be catastrophic when autophagy flux is defective. Autophagy is an important source of energy in times of stress, and a failure in degradation will eliminate this lifeline (26). When lysosomal activity is limited, excessive autophagosome synthesis is a futile action to autophagy and a wasteful process to cells, because this can further deplete energy and nutrition, thereby exerting cell toxicity. It is probable that over an extended time frame, this energy deficit stress may cause a feedback loop to induce more autophagosome formation, further exacerbating the problem and potentially promoting cell death. Because autophagy plays important roles in various elements of cellular homeostasis, other feasible contributors to the viability loss could include the failure to clear toxic substrates or the persistence of faulty and damaged organelles. Indeed, we observed an increase in ROS production following accumulation of autophagosomes, suggesting that ROS contributes to the toxicity.

A number of studies have previously demonstrated the pathological relevance of disrupting autophagy flux. Perhaps most notably, combining chemotherapeutic drugs with autophagosome clearance inhibitors has proven to increase treatment potency in a variety of tumors (25, 51–54). The rationale behind these strategies is that tumors often rely very heavily on the benefits yielded by autophagy: the nutrient supply suits their high growth demands, and it can help dissipate harmful products of their own microenvironment. Because of this, tumors often have enhanced basal autophagy rates with more autophagosome synthesis. Therefore, these malignancies may be more susceptible to autophagosome accumulation-based toxicity.

Neurodegenerative pathologies such as Alzheimer's, Parkinson's, and Huntington's are often associated with aggregation of toxic proteins. Previous studies, as well as our own data, indicate that these aggregates induce autophagosome synthesis while also disrupting autophagic degradation (25, 53, 54). It is exciting that our data indeed show that reducing accumulation of autophagosomes ameliorates cell toxicity caused by aggregation-prone proteins mHTT and α-synuclein. It is important to note that the most promising strategies for manipulating autophagy in neurodegenerative disease treatment have been those geared toward restoring flux, rather than solely elevating autophagosome synthesis (53, 55). Additionally, some previous reports have also suggested some benefit of depleting autophagy induction to neurotoxic insults (56, 57). Therefore, our model of toxicity resulting from accumulation of autophagosomes during periods of lysosome failure is entirely consistent with these studies and provides mechanistic insight into this phenomenon. The results we present indicate that a reduction in Beclin 1 is protective for cells with aggregation-prone proteins by reducing accumulation of autophagosomes. Consistent with this, Beclin 1-dependent toxicity has also been reported during ischemia/reperfusion injury in cardiomyocytes (58). In addition to Beclin 1, we also targeted another alternative regulator of autophagy, Atg16L1. Again, Atg16L1 partial depletion was also protective for cells bearing protein aggregates. Currently, autophagosome synthesis stimulation is often used to enhance autophagy to alleviate protein aggregation toxicity in neurodegeneration. Our study suggests that strategies of solely stimulating autophagosome synthesis will not be beneficial, but instead deleterious, to neurons in dementia diseases with protein aggregation.

Our findings suggest that lowering the accumulation of autophagosomes may have a therapeutic value for neurodegenerative diseases with protein aggregates. Future investigations into the toxicity of autophagosome accumulation in neurodegeneration may offer a new treatment option for these conditions.

## Experimental procedures

### Antibodies and reagents

Rabbit polyclonal antibodies were as follows: anti-mTOR 7C10 (1:500) (CST, 2983); anti-syntaxin-17 (1:1,000) (MBL, PM076); anti-LC3-II (1:10,000) (Novus Biologicals, NB100-2220); anti-Atg16L1 (1:1,000) (MBL, PM040Y); anti-Atg10 (1:1,000) (Bio-Rad, AHP1890); anti-Beclin 1 (1:1,000) (Sigma, B6061; CST, 3738); anti-caspase-3 (1:1,000) (CST, 9662); anti-cleaved caspase-3 (Asp-175) (1:1,000) (CST, 9661); anti-RIP1 (1:1,000) (CST, 3493); anti-IMPA1 (1:15,000) (Abcam, 184165); anti-SNAP29 (1:1,000) (Abcam, 138500); and anti-actin (1:2,000) (Sigma, A2066). Mouse monoclonal antibodies were as follows: anti-GAPDH (1:5,000) (Ambion, AM4300) and anti-HA (1:1,000) (Biolegend, 901501). Rap (R0395), CQ (C6628), Nec (N9037), staurosporine (STS) (S5921), and 3MA (M9281) were purchased from Sigma. Z-VAD-fmk (catalog no. 627610) was a product of Merck. PI-103 (catalog no. 528100) was from EMD4 Biosciences. Baf (catalog no. 19–148) was from Millipore. Ril was a product of Viva Bioscience (VB2706).

### Plasmids

Plasmids mRFP-GFP-LC3 (catalog no. 21074) (28), HA-α-synuclein (catalog no. 40824), and GFP-α-synuclein A53T (catalog no. 40823) (59) were obtained from Addgene. HA-tagged HTT-72Q (60), GFP-HTT-21Q, and GFP-HTT-72Q (61) were described previously. To obtain mCherry-HTT-72Q and mCherry-α synuclein, mCherry cDNA was cloned into pcDNA3 flanked with HindIII/BamHI sites. To ligate HTT-72Q into pcDNA mCherry, HTT-72Q was cut with BglII/EcoRI, and pcDNA mCherry was cut with BamHI/EcoRI. To ligate α-synuclein A53T into pcDNA mCherry, α-synuclein was cut with BamHI/XhoI, and pcDNA3 mCherry was cut with BglII/SalI. Plasmids were confirmed by DNA sequencing. Scramble shRNA (Addgene, 1864) and mTOR shRNA (Addgene, 1855) was a gift from Dr. David Sabatini (62). STX-17 shRNA was purchased from Sigma (TRCN0000379933).

### Cell culture

HeLa, HEK293, SK-N-SH, RT4-D6P2T (from Sigma, 93011415), and LAMP1/2 double knock-out or wild-type MEFs (gifts from Dr. P. Saftig, Kiel, Germany) were cultured in DMEM (D6046) medium with 10% FBS (12133C) (Sigma). mRFP-GFP-LC3–stably expressing HeLa cells, described previously (28, 60), were cultured in DMEM with 10% FBS. Human primary schwannomas were cultured as described previously (63) with full ethical approval.

Plasmids were transfected into cells either alone or with siRNA using Lipofectamine 2000 (Thermo Scientific), according to the manufacturer's instructions, for the length of time indicated in each figure legend.

### siRNA transfection

Cells were split to confluence of 60–80% and incubated in antibiotic-free DMEM (containing 10% FBS). siRNAs were transfected at a final concentration of 50 nm (unless otherwise stated), using Lipofectamine 2000 as per the manufacturer's instructions (Invitrogen). Medium was exchanged 24 h post-transfection, with cells typically incubated for a further 24–48 h before harvesting/analyzing. Human RIP1 siRNA (J-004445-07) was obtained from Dharmacon. All other siRNAs were purchased from the suppliers as indicated. Human siRNA sequences were as follows: mTOR (Invitrogen), 5′-GGGCAUGAAUCGGGAUGAU-3′ (sense); syntaxin-17 (STX-17) (Invitrogen), 5′-GACUGUUGGUGGAGCAUUU-3′ (sense); Vps33A (Invitrogen), 5′-GCAAGGCAAUAGUUUGGGA-3′ (sense); Atg10 (Invitrogen), 5′-CCAUGGGACACUAUUACGC-3′ (sense); Atg16L1 (Dharmacon, Smartpool); Beclin 1 siRNA-1 (Dharmacon), 5′-GGUCUAAGACGUCCAACAA-3′ (sense); Beclin 1 siRNA-2 (CST), 5′-GGUCUAAGACGUCCAACAA-3′ (sense); caspase-3 (CST), 5′-UGGAUUAUCCUGAGAUGGG-3′ (sense); control siRNA-1 (Dharmacon), 5′-CGUACGCGGAAUACUUCGA-3′ (sense); IMPA1 (Ambion), 5′-GGUCAAAAAUUUGGAACUU-3′ (sense); SNAP29 (Ambion), 5′-CCCACACCUUCGAGCCUAU-3′ (sense).

### Immunocytochemistry

Cells were washed with PBS twice and fixed with 4% paraformaldehyde for 10 min. Following three further PBS washes, cells were permeabilized with 0.5% Triton in PBS for 10 min. Cells were blocked in blocking buffer (1% BSA, 1% heat inactivated goat serum in PBS) for 30 min at room temperature and incubated with primary antibodies overnight at 4 °C. Cells were again washed with three 10-min PBS washes, incubated with secondary antibodies for 30 min, and then followed by an additional three 10-min PBS washes. Slides were mounted with DAPI (3 μg/ml).

### mRFP-GFP-LC3 assay

HeLa cells stably expressing mRFP-GFP-LC3 were treated with compounds or siRNAs at the indicated concentrations. After 24 h, cells were fixed in 2% paraformaldehyde for 5 min. Cellomics (Arrayscan VTI) was used to score green and red vesicles. Green vesicles are considered to be autophagosomes, and red vesicles are considered to be both autophagosomes and autolysosomes. The number of autolysosomes was obtained by subtracting the number of green vesicles from that of the red vesicles.

### Cell proliferation assay

Cell proliferation was measured with the BrdU cell proliferation assay kit (CST, 6813) following the manufacturer's instructions. Absorbance was read at 450 nm with the TECAN GENios V4.62-07/01 microplate reader (Tecan, Reading, UK) and XFLUOR4 version V 4.51 software (Tecan).

### Cell viability assay

MTT was purchased from Invitrogen (M6494). Briefly, 10 μl of a 12 mm MTT stock solution was added to culture medium and incubated at 37 °C for 4 h. Medium was exchanged with 100 μl of DMSO, mixed by pipetting, and placed on a plate shaker for 10 min. Absorbance was read at 562 nm and a reference measurement was taken at 650 nm using the TECAN GENios V4.62-07/01 microplate reader and XFLUOR4 version V 4.51 software. For each assay, the control was set as 100% viability, with all other treatments corrected against this value.

### Cytotoxicity assay

Cell cytotoxicity was measured by the CytoTox 96 non-radioactive cytotoxicity assay following the manufacturer's instructions (Promega, G1780). For this assay, all cells were cultured in 96-well plates with medium-only controls. Briefly, following the desired treatment incubation, a 50-μl volume was taken from each well and transferred to a fresh 96-well plate. From here, 50 μl of CytoTox 96 reagent was added to each well, and plates were incubated for 30 min at room temperature shielded from light. After incubation, 50 μl of stop solution was added, and absorbance was read at 490 nm using the Tecan GENios V4.62–07/01 microplate reader and XFLUOR4 version V 4.51 software.

### Flow cytometry

#### 

##### Cell death assay

Trypsinized cells were washed twice with cold PBS and resuspended in 1× binding buffer (10 mm HEPES, pH 7.4, 140 mm NaCl, 2.5 mm CaCl_2_) at 1 × 10^6^ cells/ml. 100 μl of these cells were transferred to a FACS tube, treated with 5 μl of propidium iodide (30 μg/ml) (Sigma 81845), and incubated for 15 min at room temperature. Each tube had a further 200 μl of 1× binding buffer added and then was analyzed by flow cytometry.

##### DHE staining

Cells were incubated with medium containing 10 μm dihydroethidium (Sigma, D7008) for 20 min at 37 °C and then washed twice with PBS. Next cells were trypsinized, washed with flow fluid (PBS with 2% FBS), and then resuspended in 500 μl of flow fluid. Flow cytometry was performed using the BD Biosciences Accuri C6 flow cytometer with data analyzed with the accompanying software.

### ATP assay

Intracellular ATP levels were measured with the Cell Titer-Glo luminescent cell viability assay kit (Promega, G7571) according to the manufacturer's instruction. Briefly, 100 μl of Cell Titer-Glo reagent was added to the culture medium. Cells were placed on a shaker for 5 min and then incubated at room temperature for 10 min. The SPECTRA Max M5 reader was used for luminescent reading.

### LysoSensor staining

Cells were stained with 1 μm LysoSensor Green DND-189 (Thermo Fisher, L7535) for 30 min at 37 °C. Culture medium was then replaced, and live imaging was performed via confocal microscopy at an excitation wavelength of 488 nm.

### Quantitative RT-PCR analysis

RNA was isolated with TRIzol reagent as instructed (Invitrogen). For qPCR analysis, 1 μg of RNA was reverse transcribed (Applied Biosystems, Paisley, UK) using cycles of 25 °C (10 min), 37 °C (120 min), and 85 °C (5 min). cDNA templates were then used for qPCR using the LightCycler 480 DNA SYBR Green I Master kit (Roche Applied Science) in a LightCycler 480 II system (Roche Applied Science). All primers were from Sigma and were used at 0.5 μm. Actin was used as a control to normalize the data. Actin primers were 5′-ACTGGCATCGTGATGGACTC-3′ (forward) and 5′-TCAGGCAGCTCGTAGCTCTT-3′ (reverse). mTOR primers were 5′-TAAGAAAACGGGGACCACAG-3′ (forward) and 5′-TGAGAGAAGTCCCGACCAGT-3′ (reverse). Vps33A primers were 5′-CCCACAGGACTGCAGAAGA-3′ (forward) and 5′-TCCAACTGGGAGAGAAATCG-3′ (reverse).

### Electron microscopy

Cells were fixed in 2.5% glutaraldehyde diluted in 0.1 m sodium cacodylate buffer for 2 h at room temperature. The cells were then rinsed in 0.1 m sodium cacodylate buffer, post-fixed in 1% osmium tetroxide diluted in the same buffer, rinsed with 0.1 m sodium cacodylate, and then dehydrated in an ascending ethanol series. After dehydration, the cells were embedded in agar low viscosity resin and polymerized overnight at 60 °C. Ultra-thin sections (90 nm) were cut using a Lecia EM UC7 ultramicrotome and post-stained with uranyl acetate and lead citrate. Sample sections were viewed on a JEOL 1400 transmission electron microscope (Jeol, Welwyn Garden City, UK) at 120 kV, using a variety of magnifications. For autophagosome quantification, 25 micrographs were taken with a systematic random sampling from each sample.

### Statistics

A *t* test was used, and *p* values were determined by unconditional logistical regression analysis by using the general log linear option of SPSS version 9.1 (***, *p* < 0.001; **, *p* < 0.01; *, *p* < 0.05; NS, not significant). Data from three independent experiments were analyzed (unless otherwise stated).

## Author contributions

S. L. conceived the project; R. W. B. and S. L. designed the experiments; R. W. B., S. L. R., and T. R. W. performed the experiments; S. L., R. W. B., and C. O. H. analyzed the data; and S. L. and R. W. B. wrote the paper.

## Supplementary Material

Supplemental Data

## Footnotes

2 The abbreviations used are:

TOR: target of rapamycin complex
mTOR: mammalian TOR
STX-17: syntaxin-17
Bec: beclin 1
Baf: bafilomycin A1
CQ: chloroquine
dKD: double knockdown
Z-VAD-fmk: benzyloxycarbonyl-VAD-fluoromethyl ketone
Nec: necrostatin-1
HTT: huntingtin
wtHTT: WT HTT exon 1 with 25Q
mHTT: mutant HTT exon 1 with 72Q
Syn: synuclein
Ril: rilmenidine
Rap: rapamycin
LAMP: lysosome-associated membrane protein
qPCR: quantitative PCR
IMPA: inositol monophosphatase 1
3MA: 3-methyladenine
ROS: reactive oxygen species
STS: staurosporine
MEF: mouse embryo fibroblast
MTT: 3-(4,5-dimethylthiazol-2-yl)-2,5-diphenyltetrazolium bromide
TEM: transmission electron microscopy
FSC: forward-scattered light.
