# Supplementary material for: Accumulation of autophagosomes confers cytotoxicity
Source: J Biol Chem. 2017 Jul 3;292(33):13599–614. doi: 10.1074/jbc.M117.782276 (PMC5566519; doi:10.1074/jbc.M117.782276)
Supplement: Supplemental Data [file supp_292_33_13599__index.html]

Accumulation of autophagosomes confers cytotoxicity — Accumulation of autophagosomes confers cytotoxicity — Autophagosome accumulation induces toxicity — Supplemental Data 

# Accumulation of autophagosomes confers cytotoxicity

## Supplemental Data

- supplemental text and figs (.pdf, 796 KB) - supplemental figs and legends
