# Supplementary material for: Accumulation of autophagosomes confers cytotoxicity
Source: J Biol Chem. 2017 Jul 3;292(33):13599–614. doi: 10.1074/jbc.M117.782276 (PMC5566519; doi:10.1074/jbc.M117.782276)

SUPPLEMENTAL FIGURE LEGENDS

Figure S1. Rapamycin and chloroquine synergistically induces cell viability loss
SK-N-SH cells were treated with rapamycin (Rap) (2uM), chloroquine (CQ) (25uM) or Rap + CQ for 72hrs. Cell viability was measured with MTT assay (n=4 per condition). Data are shown as mean ±sd. ***: P<0.001.

Figure S2. Dual mTOR/STX17 knockdown causes viability loss in addition to reducing cell proliferation
HEK293 cells were transfected with control, mTOR, syntaxin-17 (STX17), or mTOR+STX17 siRNA as indicated for 72hrs, then measured for cell proliferation via BrdU assay **(i)** and viability via MTT assay **(ii)**. The ratio of viability/proliferation in each treatment was calculated as corrected viability reading (without proliferation factor) **(iii)** (n=6 per condition). Data are shown as mean ±sd. *: P<0.05; ***: P<0.001.

Figure S3. Chemical induction of autophagosome synthesis and blockade of autophagosome-lysosome fusion synergistically lead to cell viability loss
A. RT4-D6P2T (RT4) cells were treated with vehicle, PI-103 (PI) (0.1 uM), Bafilomycin A1 (Baf) (10 nM) or PI+Baf for 24hrs. Cell viability was measured. Data are shown as mean±sd. **: P<0.01; ***: P<0.001. **B.** The phase-contrast images were acquired for the matching cells with the treatments as above. Scale bar: 100um. **C.** RT4 cells were treated with a series of concentrations of PI-103 alone, or with Baf (10 nM) for 24 hrs. Cell viability was measured. Data are shown as mean±sd (n=12 per condition). Data are shown as mean ±sd. **D.** RT4 cells were treated with vehicle, rapamycin (Rap) (1uM), Baf (100nM), or Rap+Baf for 24hrs. Cytotoxicity was measured using the CytoTox 96 Non-Radioactive Cytotoxicity Assay (n=5 per condition). Data are shown as mean ±sd. ***: P<0.001.

Figure S4. Increased synthesis/accumulation of non-fused autophagosomes through diverse targets widely induces cytotoxicity
A. Wild-type (Ctrl) and LAMP1/2 DKO MEFs were subjected to time course starvations as indicated. Cell viability was measured. Data are shown as mean ±sd.
B. HeLa cells stably expressing GFP-LC3 were treated with control, inositol monophosphatase 1 (IMPA), SNAP29 (SNAP) or IMPA+SNAP siRNA for 72hrs, ±chloroquine (CQ) for the last 24hrs. Cells were then fixed and imaged by confocal microscopy. The numbers of LC3 puncta per cell were quantified (n=3 repeats per condition, with at least 30 cells per repeat). Data are shown as mean ±sd. *: P<0.05; **: P<0.01; ***: P<0.001. **C.** Representative confocal images from the cells in **B** are shown. Scale bar: 20um. **D.** Human primary schwannomas were treated with CQ (25uM) and rilmenidine (Ril) (2.5uM) for 24hrs. Cell viability was measured with MTT assay. Data are shown as mean ±SEM (n=5 per condition). ***: P<0.001.

Figure S5. Inhibition of autophagosome synthesis alleviates the toxicity associated with autophagosome accumulation-inducing treatments
A. HEK293 cells were transfected with control or inositol monophosphatase 1 (IMPA) + SNAP29 (SNAP) siRNA, without or with Atg16L1 siRNA for 72hrs. Cell viability was measured with MTT assay (n=5 per condition). Data are shown as mean ±SEM, with **(i)** showing all values corrected against the overall control, and **(ii)** showing IMPA+SNAP knockdowns

corrected against their respective control. *: P<0.05; ***: P<0.001. **B.** RT4 cells were treated with a series of PI-103 (PI) concentrations with or without 3-methyladenine (3MA) (10mM) for 24hrs. Cell viability was measured. Data are shown as mean ±sd. **C.** Atg16L1+/+ or Atg16L1-/- MEFs were treated with vehicle, PI (1uM), bafilomycin-A1 (Baf) (10nM) and PI+Baf for 24hrs. Cell viability was measured. Data are shown as mean ±sd (n=12 per condition). ***: P<0.001.

Figure S6. Treatments of autophagosome induction and lysosomal inhibition cause cell death independent of apoptosis and necroptosis
A. RT4 cells were treated with vehicle, PI-103 (PI) (0.5uM), chloroquine (CQ) (25uM), zVAD (20uM) and Nec (20uM) as indicated for 20hrs. Cell death was measured with propidium iodide staining in flow cytometry (n=6 per treatment). **B.** Data are shown as mean ±sd. *: P<0.05; ***: P<0.001; ns=not significant.

Figure S7. Lowering accumulation of autophagosomes by partial depletion of autophagosome synthesis alleviates the toxicity of mutant huntingtin aggregation
A. SK-N-SH cells were transfected with RFP-HTT-21Q (wt) or RFP-HTT-72Q (mut) for 48hrs. Cells were then stained with LysoSensor Green DND-189 for 30min. Live cell images were acquired with confocal microscopy. Scale bar: 20um. **B.** SK-N-SH cells were transfected with GFP-HTT-21Q (wt) or GFP-HTT-72Q (mut) for 48hrs. Cells were then fixed, stained with DAPI and imaged with confocal microscopy. The numbers of abnormal nuclei were quantified (~200 cells per repeat, n=3 per experiment). Data are shown as mean ±sd. **: P<0.01. **C.** mRFP-GFP-LC3 cells were treated with control or Beclin 1 siRNA for 48hrs. Cells were fixed and imaged with confocal microscopy. Scale bar: 20um. **D.** The numbers of autophagosomes (green vesicles) and autolysosomes (red vesicles minus green vesicles) from **C** were collected (n=50 cells per condition). Data are shown as mean ±sem. ***: P<0.001. **E.** SK-N-SH cells were transfected with GFP-HTT-21Q (wt) or GFP-HTT-72Q (mut) and either control or Beclin1 siRNA (20nM) for 48hrs. Cells were then fixed, stained with DAPI and imaged with confocal microscopy. The numbers of abnormal nuclei were quantified (~200 cells per repeat, n=3 per experiment). Data are shown as mean ±sd. *: P<0.05; **: P<0.01.

Figure S8. Mutant synuclein does not cause lysosome deacidification
SK-N-SH cells were transfected with RFP (Ctrl) or RFP-Syn-A53T for 48hrs. Cells were then stained with LysoSensor Green DND-189 for 30min. Live cell images were acquired with confocal microscopy. Scale bar: 20um.

Fig. S1

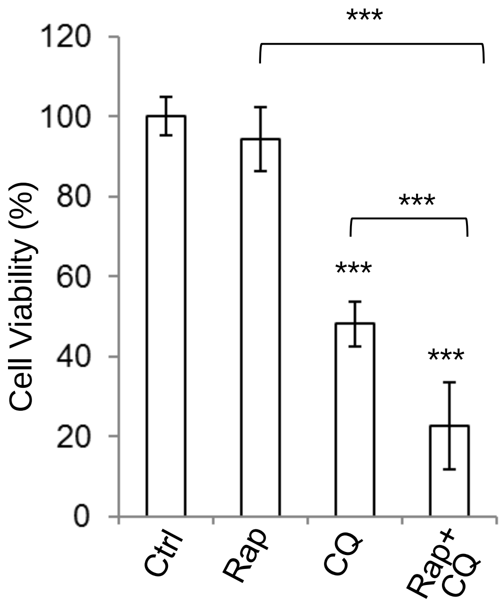

Fig. S2

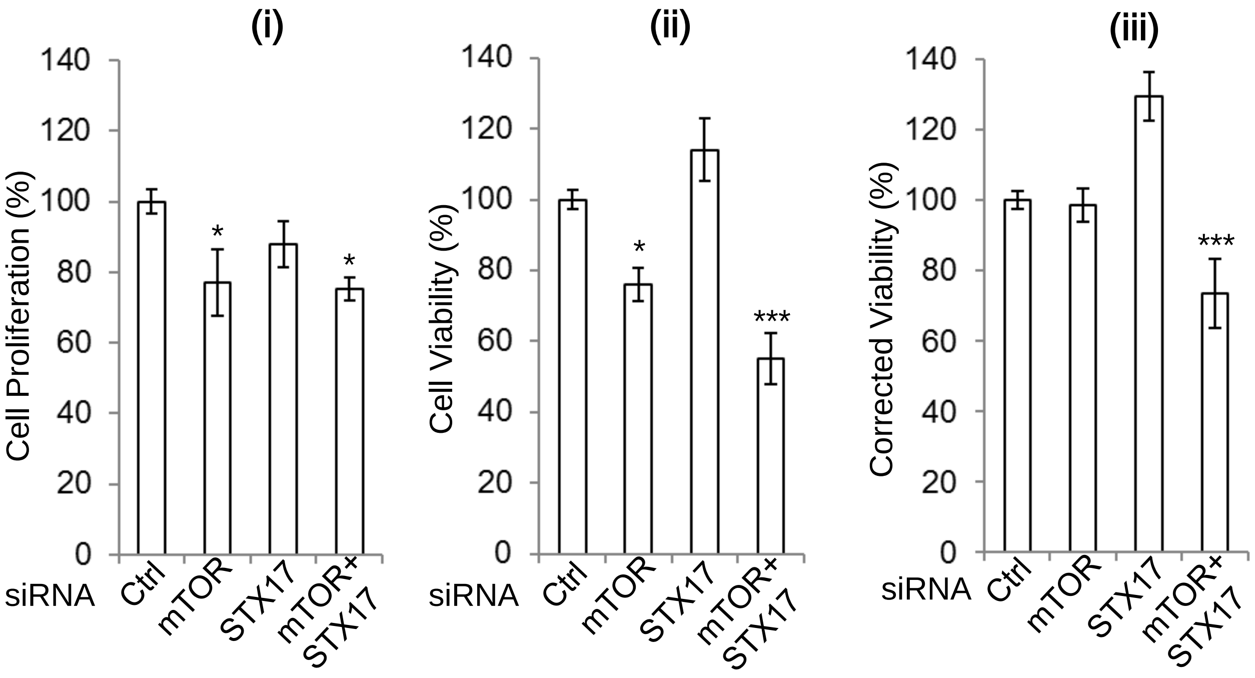
(i)
Cell Proliferation (%)
140
120
100
80
60
40
20
0
*
*
siRNA
Ctrl
mTOR
STX17
mTOR+ STX17
(ii)
Cell Viability (%)
140
120
100
80
60
40
20
0
*
***
siRNA
Ctrl
mTOR
STX17
mTOR+ STX17
(iii)
Corrected Viability (%)
140
120
100
80
60
40
20
0
***
siRNA
Ctrl
mTOR
STX17
mTOR+ STX17

Fig. S3

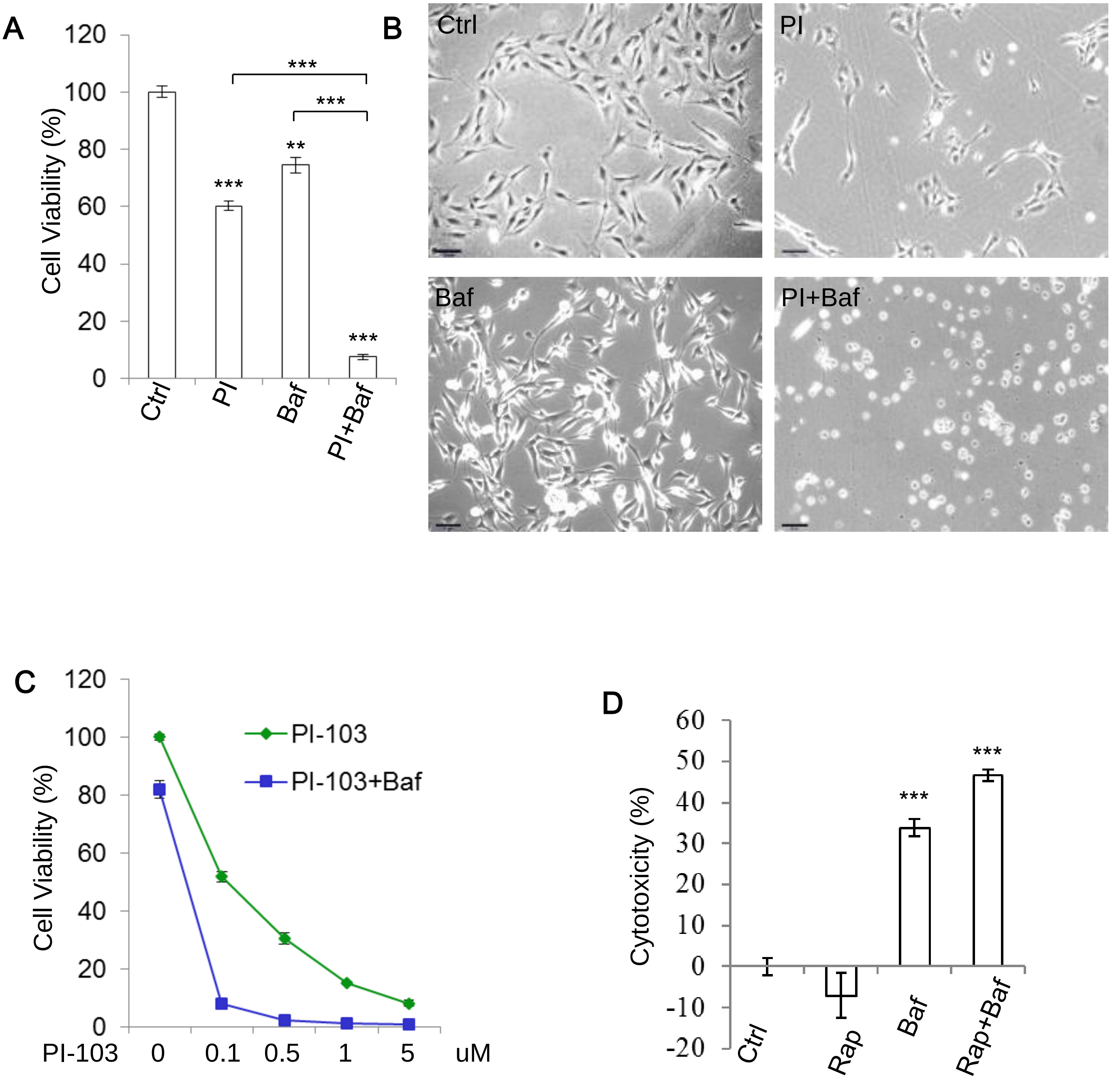

A
B
C
D
Cell Viability (%)
120
100
80
60
40
20
0
***
**
Ctrl
PI
Baf
PI+Baf
PI-103
PI-103+Baf
0
0.1
0.5
1
5
uM
Cytotoxicity (%)
60
50
40
30
20
10
-10
-20
Rap
Rap+Baf

Fig. S4

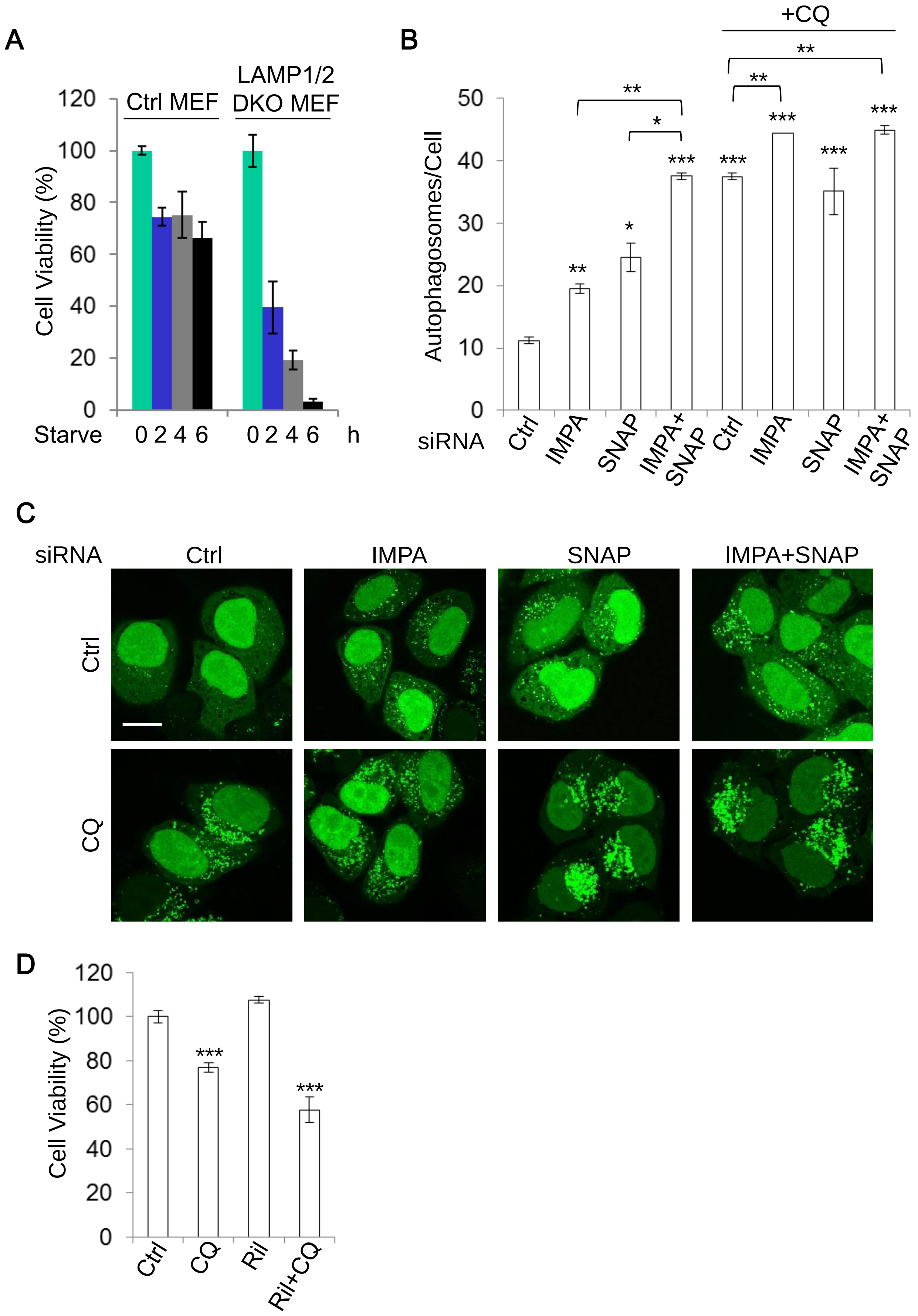
A
Cell Viability (%)
120
100
80
60
40
20
0
Ctrl MEF
LAMP1/2
DKO MEF
Starve
0 2 4 6
0 2 4 6
h
B
+CQ
Autophagosomes/Cell
50
40
30
20
10
0
siRNA
Ctrl
IMPA
SNAP
IMPA+
SNAP
Ctrl
IMPA
SNAP
IMPA+
SNAP
C
siRNA
Ctrl
IMPA
SNAP
IMPA+SNAP
Ctrl
CQ
D
Cell Viability (%)
120
100
80
60
40
20
0
Ctrl
CQ
Ril
Ril+CQ

Fig. S5

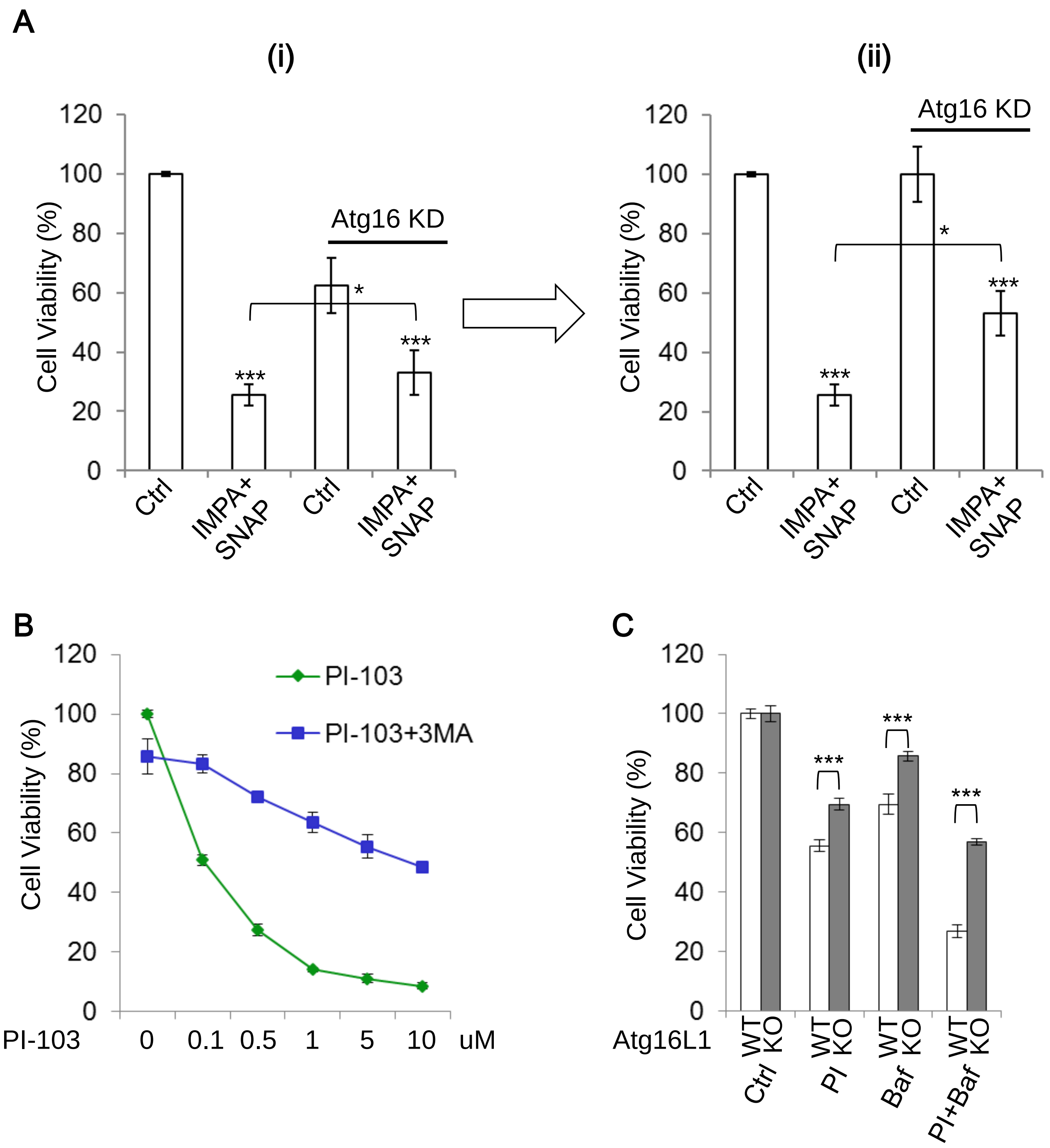
A
(i)
(ii)
Atg16 KD
Cell Viability (%)
Ctrl
IMPA+ SNAP
*
***
B
PI-103
PI-103+3MA
0
0.1
0.5
1
5
10
uM
C
Atg16L1
WT
KO
Ctrl
PI
Baf
PI+Baf

Fig. S6

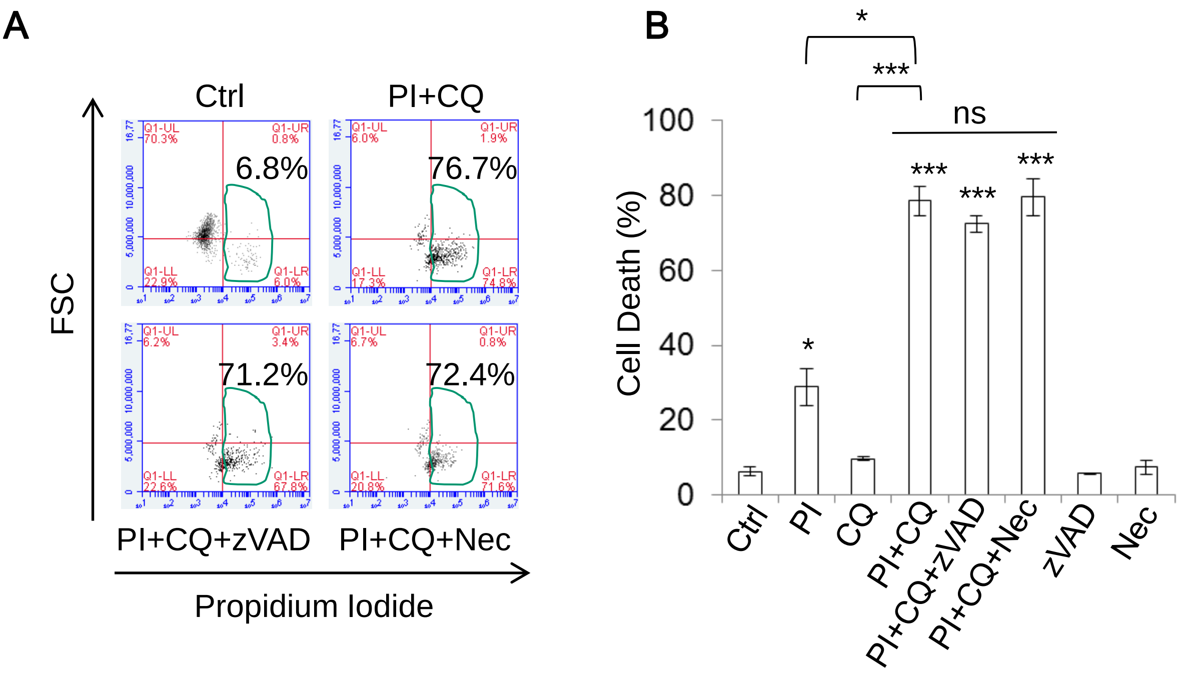
A
Ctrl
PI+CQ
6.8%
76.7%
71.2%
72.4%
PI+CQ+zVAD
PI+CQ+Nec
FSC
Propidium Iodide
B
Cell Death (%)
100
80
60
40
20
0
ns
Ctrl
PI
CQ
PI+CQ
PI+CQ+zVAD
PI+CQ+Nec
zVAD
Nec

Fig. S7

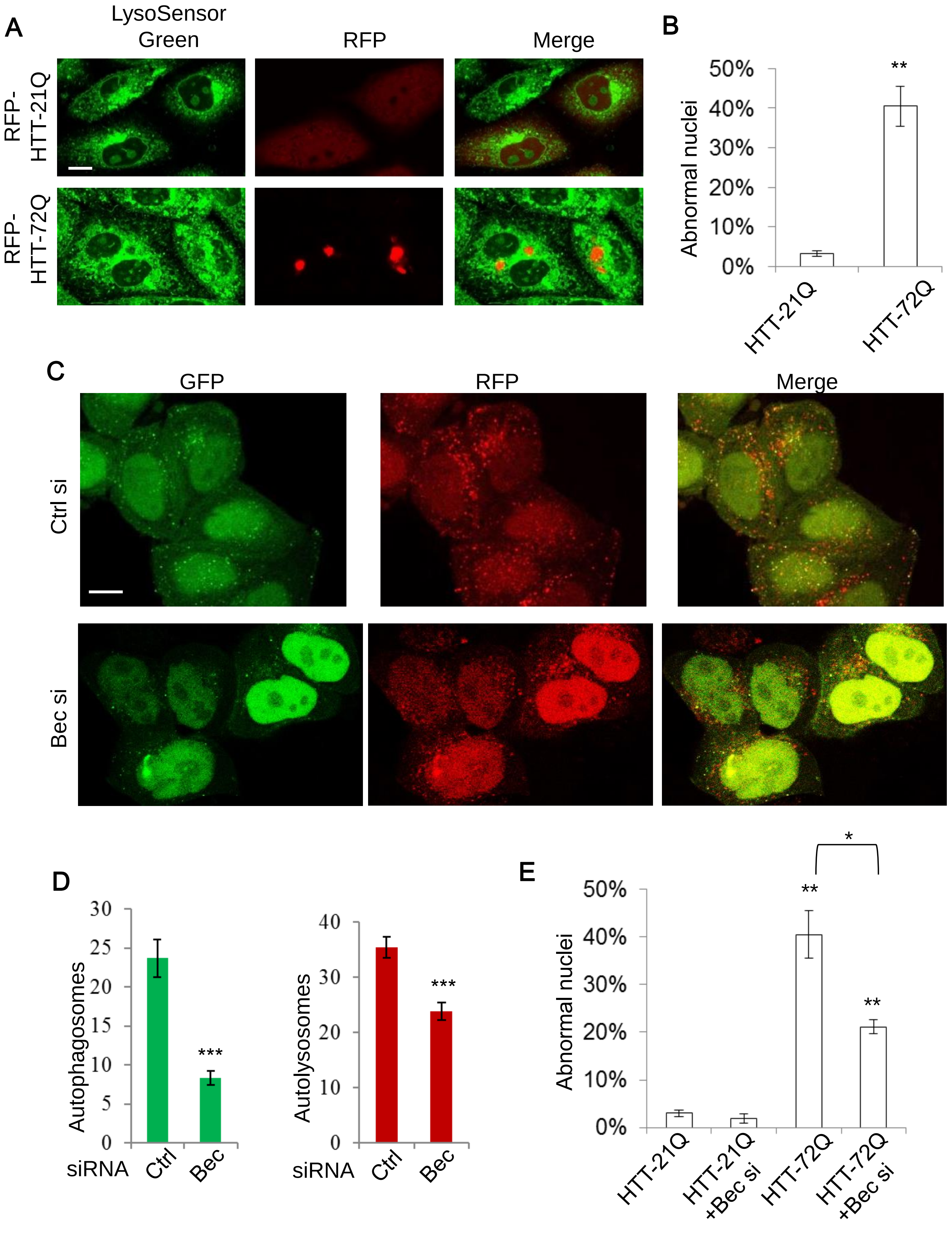
A
LysoSensor Green
RFP
Merge
RFP-HTT-21Q
RFP-HTT-72Q
B
Abnormal nuclei
50%
40%
30%
20%
10%
0%
**
HTT-21Q
HTT-72Q
C
GFP
RFP
Merge
Ctrl si
Bec si
D
Autophagosomes
30
25
20
15
10
5
0
***
siRNA
Ctrl
Bec
Autolysosomes
40
30
20
10
0
***
siRNA
Ctrl
Bec
E
Abnormal nuclei
50%
40%
30%
20%
10%
0%
*
**
**
HTT-21Q
HTT-21Q +Bec si
HTT-72Q
HTT-72Q +Bec si

Fig. S8

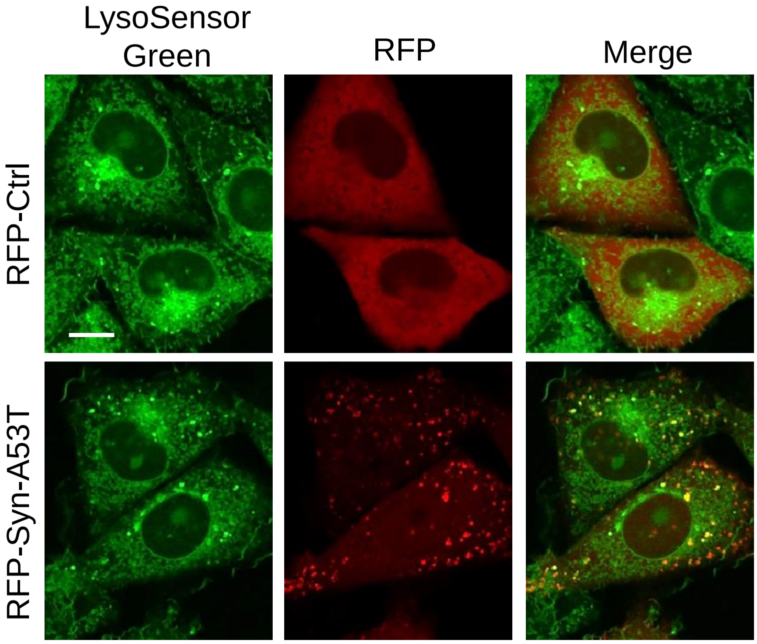

Supplement: Supplemental Data [file 10.1074_M117.782276_jbc.M117.782276-1.pdf]
